# Origins and biogeography of the *Anolis crassulus* subgroup (Squamata: Dactyloidae) in the highlands of Nuclear Central America

**DOI:** 10.1186/s12862-017-1115-8

**Published:** 2017-12-21

**Authors:** Erich P. Hofmann, Josiah H. Townsend

**Affiliations:** 1 Department of Biology, Indiana University of Pennsylvania, Indiana, PA 15705-1081 USA; 2 Present Address: Department of Biological Sciences, Clemson University, Clemson, SC 29634 USA

**Keywords:** Anoles, Chortís Block, Cryptic lineages, Divergence dating, Evolution, Multilocus phylogenetics, Reptilia, *Norops* clade

## Abstract

**Background:**

Recent studies have begun to reveal the complex evolutionary and biogeographic histories of mainland anoles in Central America, but the origins and relationships of many taxa remain poorly understood. One such group is the *Anolis (Norops) crassulus* species subgroup, which contains ten morphologically similar highland taxa, the majority of which have restricted distributions. The nominal taxon *A. crassulus* has a disjunct distribution from Chiapas, Mexico, through Guatemala, in the highlands of El Salvador, and in the Chortís Highlands of Honduras. We test the relationships of these species using multiple mitochondrial and nuclear loci in concatenated and multispecies coalescent frameworks, in an effort to both resolve long-standing taxonomic confusion and present new insights into the evolution and biogeography of these taxa.

**Results:**

Sequences of multiple mitochondrial and nuclear loci were generated for eight of the ten species of the *Anolis crassulus* species subgroup. We analyzed phylogenetic relationships and estimated divergence times and ancestral ranges of the subgroup, recovering a monophyletic subgroup within *Anolis*. Within the nominal taxon *Anolis crassulus,* we recovered multiple genetically distinct lineages corresponding to allopatric populations, and show that the Chortís Highland lineage split from the others over 13 MYA. Additionally, distinct mitochondrial lineages are present within the taxa *A. heteropholidotus* and *A. morazani,* and importantly, samples of *A. crassulus* and *A. sminthus* previously used in major anole phylogenetic analyses are not recovered as conspecific with those taxa*.* We infer a Chortís Highland origin for the ancestor of this subgroup, and estimate cladogenesis of this subgroup began approximately 22 MYA.

**Conclusions:**

Our results provide new insights into the evolution, biogeography, and timing of diversification of the *Anolis crassulus* species subgroup. The disjunctly distributed *Anolis crassulus* *sensu* *lato* represents several morphologically conserved, molecularly distinct anoles, and several other species in the subgroup contain multiple isolated lineages.

**Electronic supplementary material:**

The online version of this article (doi: 10.1186/s12862-017-1115-8) contains supplementary material, which is available to authorized users.

## Background

Anoline lizards are a well-known focal system for studying mechanisms and drivers of evolution [1, 2]. Studies of the replicated adaptive radiations of anoles on Caribbean islands [3] have greatly expanded our understanding of evolution and biogeographic patterns (e.g. [4–6]). In contrast to their island counterparts, mainland anoles have been relatively understudied, despite hosting the majority of the diversity of these lizards [7]. Recent studies of South American anoles have provided a clearer understanding of the relationships and biogeography of those taxa (e.g. [8–11]). Central America has been a setting for biogeographic studies of a variety of taxa, including snakes (e.g. [12, 13]), mammals (e.g. [14]), fish (e.g. [15]), and amphibians (e.g. [16]). Only recently, however, have studies begun to focus on the evolutionary history, diversity, and biogeography of Central American anoles, revealing a not wholly unexpected degree of complexity in patterns relating to these lizards (e.g. [17, 18]). It is apparent that the diversity of mainland anoles has been greatly underestimated, and that comprehensive work remains across virtually all mainland taxa.

**Fig. 1:**
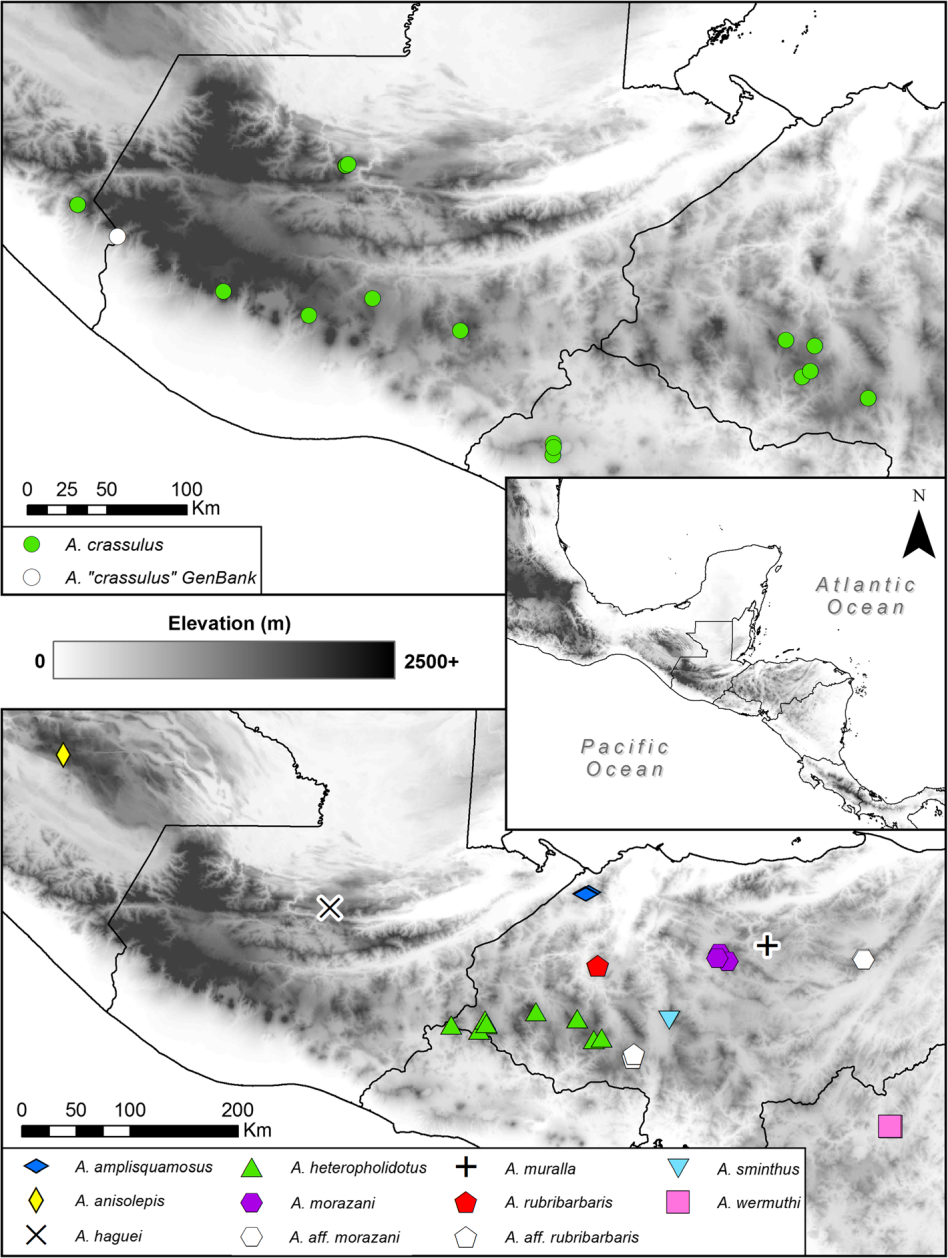
Sampling localities of the *Anolis crassulus* species subgroup: *A. crassulus* *sensu* *lato* (top) and the remaining subgroup species (bottom). The type localities of two subgroup taxa, *A. haguei* and *A. muralla,* are also noted for completeness, though they were not able to be included in this study

One particularly poorly resolved group of mainland anoles is the *Anolis (Norops) crassulus* subgroup, consisting of ten nominal species known from intermediate-to-high elevations (1000–2500 m above sea level) in Central America: *A. amplisquamosus, A. anisolepis, A. crassulus, A. haguei, A. heteropholidotus, A. morazani, A. muralla, A. rubribarbaris, A. sminthus,* and *A. wermuthi* (*sensu * [19]). Anoles referred and bearing an affinity to *A. crassulus* have long been recognized as needing thorough resolution of their evolutionary relationships and taxonomy [19–22]. Seven of the ten species are endemic to the Chortís Block Highlands of Honduras and adjacent El Salvador and Nicaragua [19, 23, 24]. *Anolis anisolepis* and *A. haguei* are known only from Chiapas, Mexico, and Alta Verapaz, Guatemala, respectively [19, 23], and are of questionable taxonomic validity [25]. Populations assigned to the nominal taxon *A. crassulus* can be found from southwestern Honduras to Chiapas, Mexico at elevations between 1300 and 3000 m [19, 23]. However, *A. crassulus* has a disjunct distribution across Central America, with isolated populations ranging in Chiapas, Mexico, southern El Salvador, across central Guatemala, and western Honduras (Fig. 1) [23]. These species all inhabit a similar ecological niche largely restricted to Lower Montane Wet and Moist Forest formations, and are found active on the ground and low vegetation or tree trunks during the day and asleep on low vegetation at night [19].

This subgroup has long been a taxonomically confusing complex, and past studies using only morphological examinations have failed to resolve relationships of named taxa and populations. Meyer and Wilson ([20]: 108) stated “specimens of the *crassulus* group from Guatemala and Mexico have a bewildering array of admixtures of the distinctive characters observed in Honduras… The inter-relationships of [this subgroup] are exceedingly complex, and… we are unable to suggest a satisfactory arrangement.” Subsequent authors, including Lieb [21], McCranie et al. [22], and McCranie and Köhler [19] have commented on the importance of a deeper investigation into this subgroup. Previously, these anoles been divided into ‘*crassulus-*like’ anoles (*Anolis anisolepis, A. crassulus, A. haguei, A. morazani,* and *A. rubribarbaris*) and ‘*sminthus-*like’ anoles (*A. heteropholidotus, A. muralla, A. sminthus,* and *A. wermuthi*) on the basis of morphological similarities [24, 26]. As noted by Townsend and Wilson [24], the relationship of *Anolis amplisquamosus* to the *A. crassulus* subgroup remains poorly understood. The validity of *A. anisolepis* and *A. heteropholidotus* have been questioned, and in some cases, these taxa are still not recognized. Köhler [27] demonstrated the validity of *A. heteropholidotus*, which has subsequently been recognized by Townsend and Wilson [24], Johnson et al. [28], McCranie and Köhler [19], and Poe et al. [29]. However, Lieb [25] stated that *A. heteropholidotus* was not distinct at the species level, Nicholson et al. [30] did not list *A. heteropholidotus* as a valid species, and both the IUCN [31] and Uetz et al. [32] continue to regard *A. heteropholidotus* as a synonym for *A. sminthus*. *Anolis anisolepis* was considered a valid species by Nicholson et al. [30], but not by Poe et al. [29].

Despite the poor understanding of the relationships within the *Anolis crassulus* species subgroup and recent increased sampling effort, the majority of previous anole phylogenies have included, at most, two samples of the *Anolis crassulus* subgroup: a sample considered to represent *A. crassulus* from Chiapas, Mexico, and another sample identified as *A. sminthus* from Olancho, Honduras. As this study was being finalized, two major anole phylogenies became available that expanded the sampling of this group: Nicholson et al. [18] and Poe et al. [29]. Nicholson et al. [18] used a continuous 1423 bp sequence of mitochondrial DNA to analyze the biogeography of mainland *Anolis*. They included a single sample of *A. crassulus, A. amplisquamosus, A. wermuthi, A. heteropholidotus, A. morazani,* and *A. sminthus,* and recovered them as a monophyletic subgroup within their larger “*A. crassulus* clade”*.* The *A. crassulus* specimen, MZFC 6458 from Chiapas, Mexico, was the same sequence used in Nicholson et al. [7, 18], and sampled from the same specimen sequenced by Gray et al. [33] under a different GenBank accession number. The *A. sminthus* sample, SMF 78830 from Olancho, Honduras, was the same sequence used in Nicholson et al. [7, 18]. McCranie & Köhler [19] noted that that SMF 78830 is not actually a specimen of *A. sminthus,* and opined that it likely represents an undescribed species more closely related to *A. crassulus.* Poe et al. [29] included molecular samples (at least two mitochondrial and/or nuclear loci) of *A. crassulus, A. amplisquamosus,* and *A. sminthus,* and scored *A. heteropholidotus, A. morazani, A. muralla, A. rubribarbaris,* and *A. wermuthi* for morphological characters only (no molecular data). In their combined phylogeny, Poe et al. [29] did not recover a monophyletic *A. crassulus* subgroup, and the placement of the taxa scored only for morphology was not well supported.

The goals of this study were to resolve the evolutionary histories of taxa and populations assigned to the *Anolis crassulus* subgroup, and determine the subgroup’s biogeographic origins and patterns of diversification in Central America. We assembled and analysed a multilocus phylogenetic dataset (three mitochondrial loci and three nuclear loci) of the majority of ingroup taxa (including multiple samples from across their distributions, when available), and estimated divergence times and ancestral distributions to provide a historical context for their diversification. We provide new insights into the evolutionary history of these taxa in an attempt to resolve some of the confusion that has plagued this group for decades.

## Methods

### Taxon sampling

Most samples used in this study were collected across Honduras and northern Nicaragua by JHT and colleagues between 2005 and 2015 (Fig. 1). Prior to formalin-fixing specimens, a tissue sample was removed and preserved in SED buffer (20% DMSO, 0.25 M EDTA, pH 7.5, NaCl saturated; [34–36]). Samples of *Anolis anisolepis* and additional samples of *A. crassulus* and *A. heteropholidotus* were received from the University of Kansas Biodiversity Institute (KU; Lawrence, Kansas, USA), the Museum of Vertebrate Zoology (MVZ; Berkeley, California, USA), and the Senckenberg Forschungsinstitut und Naturmuseum (SMF; Frankfurt am Main, Germany). Institutional abbreviations follow those standardized by the American Society of Ichthyologists and Herpetologists [37]. A list of all samples and their localities can be found in Additional file 1: Appendix 1.

### DNA sequence generation

We generated sequences of three mtDNA loci and three nDNA loci for eight of the ten ingroup species (tissues of *Anolis haguei* and *A. muralla* were not available) and three outgroup taxa (*A. cusuco, A. kreutzi,* and *A. laeviventris* from Honduras; the *A. laeviventris* species subgroup * sensu * [19]). DNA extraction, amplification, and sequencing of mitochondrial genes 16S large subunit rRNA (16S) and cytochrome oxidase subunit I (COI) of some samples were carried out at the Smithsonian Institution Laboratory of Analytical Biology (LAB; Suitland, Maryland, USA) following standardized DNA Barcode of Life (BOLD) protocols. Template DNA was extracted by an AutoGen Geneprep 965 (Autogen, Holiston, MA) via a phenol-chloroform technique. The two mitochondrial genes were amplified via polymerase chain reaction (PCR). Two μl of ExoSAP-IT (Affymetrix, Santa Clara, CA) was used to remove unincorporated nucleotides from the PCR product, which was sequenced using a BigDye Terminator v3.1 Cycle Sequencing kit (ThermoFisher, Waltham, MA), cleaned using Sephadex spin column filtration, and electrophoresed on an ABI 3730xl DNA Analyzer.

All other sequences used herein, including additional loci generated for samples sequenced for only 16S and COI at LAB, were generated in the Townsend Lab at Indiana University of Pennsylvania (Indiana, PA, USA). Whole-genome DNA was extracted from tissue using PureLink Genomic DNA Kits (ThermoFisher). All available samples were first amplified for 16S and COI via PCR. A subsample of these was then amplified for an additional mitochondrial locus, NADH dehydrogenase subunit 2 (ND2), and three nuclear loci: prolactin receptor (PRLR), brain-derived neutrophic factor (BDNF), and protein tyrosine phosphatase non-receptor type 12 (PTPN12). PRLR and PTPN12 are considered relatively variable nuclear markers in squamate reptiles, while BDNF is considered relatively conserved [38].

PCR conditions varied between the six genes, but were carried out in 25 μl reactions (20 μl for 16S and PRLR) containing concentrations of 1× PCR buffer, 1.5–2 mM MgCl_2_, 0.3–0.875 mM dNTPs, 0.8–2.5 μM of each primer (forward and reverse), 0.05 U Taq polymerase, and 1–2 μl of sample (~25 ng/μl). Samples that failed to amplify using the standard conditions were attempted again in a 25 μl reaction containing 12.5 μl of 1× Platinum SuperFi Green PCR Master Mix (ThermoFisher), 1.25 μl of each primer (0.5 μM concentration), 2 μl of sample (~25 ng/μl), and 8 μl of H_2_O. All primers and cycling parameters used in this study are available in Additional file 1: Appendix 2.

Unincorporated nucleotides were removed from all PCR products using 2 μL of ExoSAP-IT (Affymetrix) per sample, and products were sent to Eurofins Scientific (Louisville, Kentucky, USA) for sequencing. They were electrophoresed on an ABI 3730xl DNA Analyzer. Chromatograms were checked manually and sequences were assembled using Geneious v.7.1.7 [39]. Sequence alignment was carried out in MEGA7 [40] using the ClustalW algorithm [41], and redundant identical haplotypes were removed to streamline analyses. Transfer RNAs (tRNAs) at the ends of the ND2 sequences were removed due to inconsistencies in the sequencing of those regions. All sequences were deposited on GenBank (Additional file 1: Appendix 1). Additional ND2 sequences were pulled from GenBank (Additional file 1: Appendix 3) and analyzed with the ND2 sequences generated in this study.

Four datasets were then created from the full sequence data: ND2 only (75 samples), all mtDNA (16S + ND2 + COI; 43 samples), all nDNA (PRLR + BDNF + PTPN12; 37 samples), and all six loci concatenated (37 samples). The ND2 dataset included samples from GenBank and was analysed separately to determine if the *Anolis crassulus* subgroup is monophyletic with respect to other anoles*.* An additional ND2 dataset (19 samples) was used for divergence dating analysis (see below).

### Phylogenetic analyses

In all analyses, unless otherwise stated, the datasets were partitioned by gene for 16S and codon position for the five protein-coding loci (ND2, COI, PRLR, BDNF, PTPN12). Best fit models of nucleotide substitution (Additional file 1: Appendix 4) were selected for each partition using PartitionFinder v.1.1.1 [42] via the Akaike information criterion (AIC), a greedy search scheme, and limiting the substitution models to those that could be implemented in MrBayes 3.5.2 [43, 44]. The ND2-only datasets (one including a larger sample of all *Anolis* and one including the *A. chlorocyanus* group; see below) were analysed separately to account for the additional sequences from GenBank.

Maximum likelihood (ML) analyses were carried out in RAxML v7.2.8 [45] using raxmlGUI v1.5 [46], with 1000 bootstrap pseudoreplicates under the default GTR-GAMMA substitution model. Bayesian inference (BI) was performed using MrBayes 3.5.2 [43, 44], and consisted of two parallel runs of four Markov chains (three heated, one cold) run for 20 × 10^6^ generations and sampled every 10,000 generations, with a random starting tree and the first 2 × 10^6^ generations discarded as burn-in. The resulting topologies were visualized using FigTree v1.4.2 [47] and annotated using Inkscape v0.91 [48].

To account for potential incongruences between mitochondrial-only and nuclear-only datasets due to incomplete lineage sorting, we inferred the species tree of our dataset under a Bayesian multispecies coalescent framework in StarBEAST2 [49], implemented in BEAST 2.4.7 [50]. Use of the multispecies coalescent framework have been shown to produce accurate estimates of species trees from independent gene trees, even when gene trees are not congruent [51, 52]. This analysis requires a priori assignment of individuals to “species”, which was done based on the results of our prior analyses. Two individuals per species were included when possible, though we included only samples that had at least two loci sequenced and some taxa had only single exemplars; in total, 35 individuals were used. We did not phase our nuclear data as many outgroup and ingroup taxa had only single representative individuals. We partitioned our dataset by locus, unlinking substitution models across each (based on PartitionFinder v1.1.1. ran as above, except limiting the models to those implementable in BEAST; Additional file 1: Appendix 4), and linking molecular clock and tree priors in the three mitochondrial loci while leaving them unlinked in the three nuclear genes. Four independent analyses were performed using a strict clock and Yule tree prior, for 100 million generations, sampling every 5000. We examined the resulting logfiles in Tracer v1.6 [53] to ensure effective sample sizes (ESS) were above 200, and combined trees and log files in LogCombiner v.2.4.7 [50], removing a burn-in of 10%. Finally, the combined tree files were annotated with TreeAnnotator v2.4.7 [50], using mean heights to calculate the maximum clade credibility (MCC) tree.

### Divergence dating

We estimated the timing of diversification of the *Anolis crassulus* subgroup in BEAST 1.7.5 [54]. We analyzed the ND2 sequences from one representative of each ingroup lineage where possible (13 samples; Additional file 1: Appendix 1), with an uncorrelated lognormal relaxed clock model and a Yule tree prior. Initially, we chose only the calibration rate of 0.65% per lineage per million years (MY) [55]. This rate was calculated from approximately 1700 bp of ND1, ND2, and COI, including transfer RNAs [55], and has been used in several phylogenetic studies of anoles without fossil calibrations to determine the approximate timing of various taxa (e.g. [8, 11, 17, 56]). However, as our ND2 sequences did not cover the entirety of Macey et al. [55]’s sequences, including the conserved trailing tRNAs, the 0.65%/MY rate alone greatly overestimated divergence dates in comparison to recent phylogenies [8, 29, 30, 57]. Analyses using the published rate in addition to a fossil calibration (see below) continued to produce dates older than other recent phylogenies. Application of a calibration rate of 0.765% per lineage per MY (approximately 15% faster, accounting for the lack of conserved tRNAs in our sequences) estimated dates more comparable (though not identical) to those of previous studies [8, 29, 30, 57], and was chosen for continued analysis together with a fossil calibration in order to provide a general temporal context for the diversification of this subgroup. As there are no known fossils within the *A. crassulus* subgroup, we added four ND2 sequences of the *A. chlorocyanus* species group (*A. alinger, A. chlorocyanus, A. coelestinus,* and *A. singularis*; Additional file 1: Appendix 1) from GenBank to our dataset, allowing one fossil calibration utilized in major anole phylogenies [29, 30] to be incorporated: a Dominican amber anole assigned to this species group [58]. Following Poe et al. [29], we placed this calibration on the most recent common ancestor node of the *A. chlorocyanus* species group, and used a uniform prior distribution based on the stratigraphic information of the fossil (17–23 MYA).

We implemented this dating method in order to determine a general historical context for the diversification of the subgroup and report the resulting values cautiously, given they are estimated from a single mitochondrial locus with an approximated rate, and a single fossil calibration. Four independent runs were performed in BEAST with the same number of generations and frequency of sampling as the species tree analysis. As with the species tree analysis, the resulting log files were examined in Tracer v1.6 [53], log and tree files were combined in LogCombiner v.1.7.5 [54] and combined tree files were annotated with TreeAnnotator v1.7.5 [54].

### Ancestral area reconstruction

To infer the ancestral distribution of the *Anolis crassulus* subgroup, we conducted ancestral area reconstruction using a Bayesian Binary Markov-chain Monte Carlo (BBM) [59] analysis implemented in RASP v3.2 [60] using the post burn-in trees obtained from BEAST to account for phylogenetic uncertainty. This method statistically infers an ancestral state at a node by averaging its frequency across all trees, using a full hierarchical Bayesian approach and hypothesizing a special distribution, the “null distribution”, where an ancestral area does not contain any of the unit areas [60]. We assigned each ingroup taxon to one of four biogeographical regions: (A) Chiapas, Mexico; (B) Guatemala; (C) Salvadoran Cordillera; (D) Chortís Highlands. These boundaries isolate all of the ingroup taxa analyzed herein, and represent biogeographic breaks corresponding to speciation in reptiles (e.g. [12, 13]). Outgroup taxa were removed from the analysis. The maximum potential areas occupied was set to four, the Fixed Jukes-Cantor model (with gamma shape parameter) was chosen, and the analysis was run for 1 million cycles using 10 chains, sampling every 100 generations and discarding the first 10% as burn-in.

## Results

### Sampling success

In total, 107 samples of ingroup taxa were sequenced for 16S (444 bp) and 104 samples for COI (654 bp). This group was then subsampled to representatives of the recovered lineages, but due to problems with PCR amplification and/or gene sequencing, some samples failed to properly amplify for certain genes. Twenty-three samples of ingroup taxa were amplified for ND2 (1038 bp), 39 for PRLR (545 bp), 31 for BDNF (658 bp), and 25 for PTPN12 (832 bp), giving a final combined dataset of 4171 bp (2136 bp mtDNA, 2035 bp nDNA).

### Phylogenetic relationships

All phylogenetic analyses recovered a monophyletic *Anolis crassulus* species subgroup, with multiple lineages assigned to some nominal taxa. The previous assignment of species to “*crassulus*-like” and “*sminthus*-like” series within the subgroup was not supported. Instead, in most analyses we recovered a clade containing *A. anisolepis* and *A. crassulus* *sensu* *lato* (hereafter the *A. crassulus* clade) as sister to a clade that contained most of the Chortís Highland endemics: *A. sminthus, A. morazani* *sensu* *lato, A. rubribarbaris,* and *A. heteropholidotus* *sensu* *lato* (hereafter the *A. sminthus* clade). *Anolis* aff. *rubribarbaris* and *A. wermuthi* were also recovered as part of this clade when included in mitochondrial only and species tree analyses. The placement of *A. amplisquamosus* was problematic and poorly supported across all analyses.

**Fig. 2:**
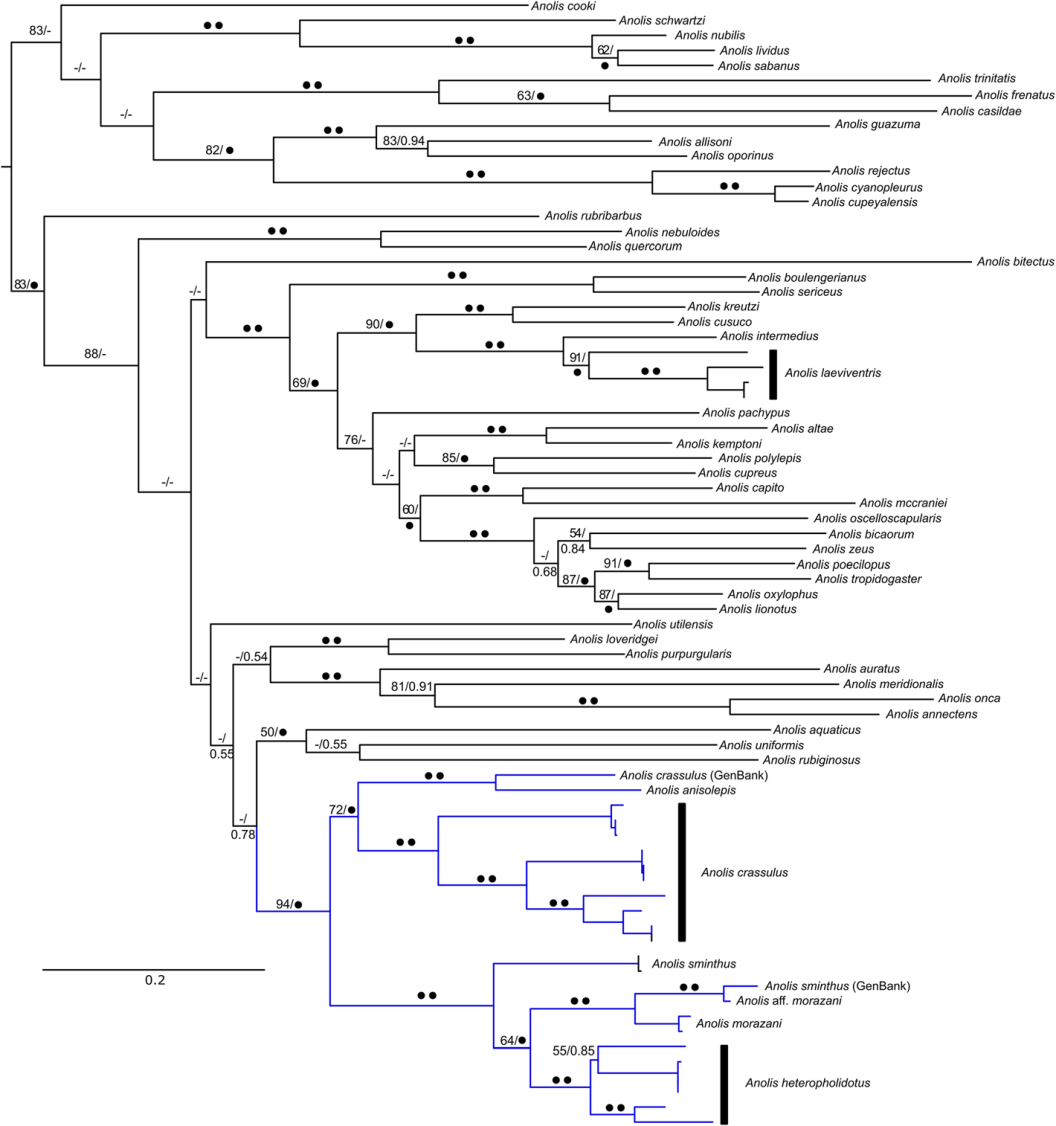
Full ML topology based on mtDNA locus ND2, showing the placement of the *Anolis crassulus* subgroup (blue) within a larger sampling of the *Anolis* clade. Nodes are labelled with maximum likelihood bootstrap support (BS; left) and Bayesian inference posterior probabilities (PP; right). Black circles indicate BS ≥ 95 or PP ≥ 0.95. BS < 50 and PP < 0.50 not shown

Analyses of the larger ND2 dataset (Fig. 2) recovered the *Anolis crassulus* subgroup as a well-resolved clade within the rest of *Anolis*. It is important to note that MZFC 6458, the sample used to represent *A. crassulus* in previously published anole phylogenies, was not recovered with any other samples of *A. crassulus,* including those from nearby localities in Chiapas, Mexico, and was instead recovered as a sister lineage to *A. anisolepis.* Additionally, SMF 78830, the sample of *A. “sminthus”* referred to as a potentially unrecognized species related to *A. crassulus* by McCranie & Köhler [19], was recovered as part of a lineage with other *“crassulus”-*like anoles from Olancho that is sister to *A. morazani.* Based on this information, we infer that the samples referred to herein as *A.* aff. *morazani* are conspecific with McCranie and Köhler [19]’s “*crassulus-*like” population from Olancho. Further investigation of this population is ongoing. Unfortunately, MZFC 6458 and SMF 78830 were not available to be sequenced for the remaining loci.

**Fig. 3:**
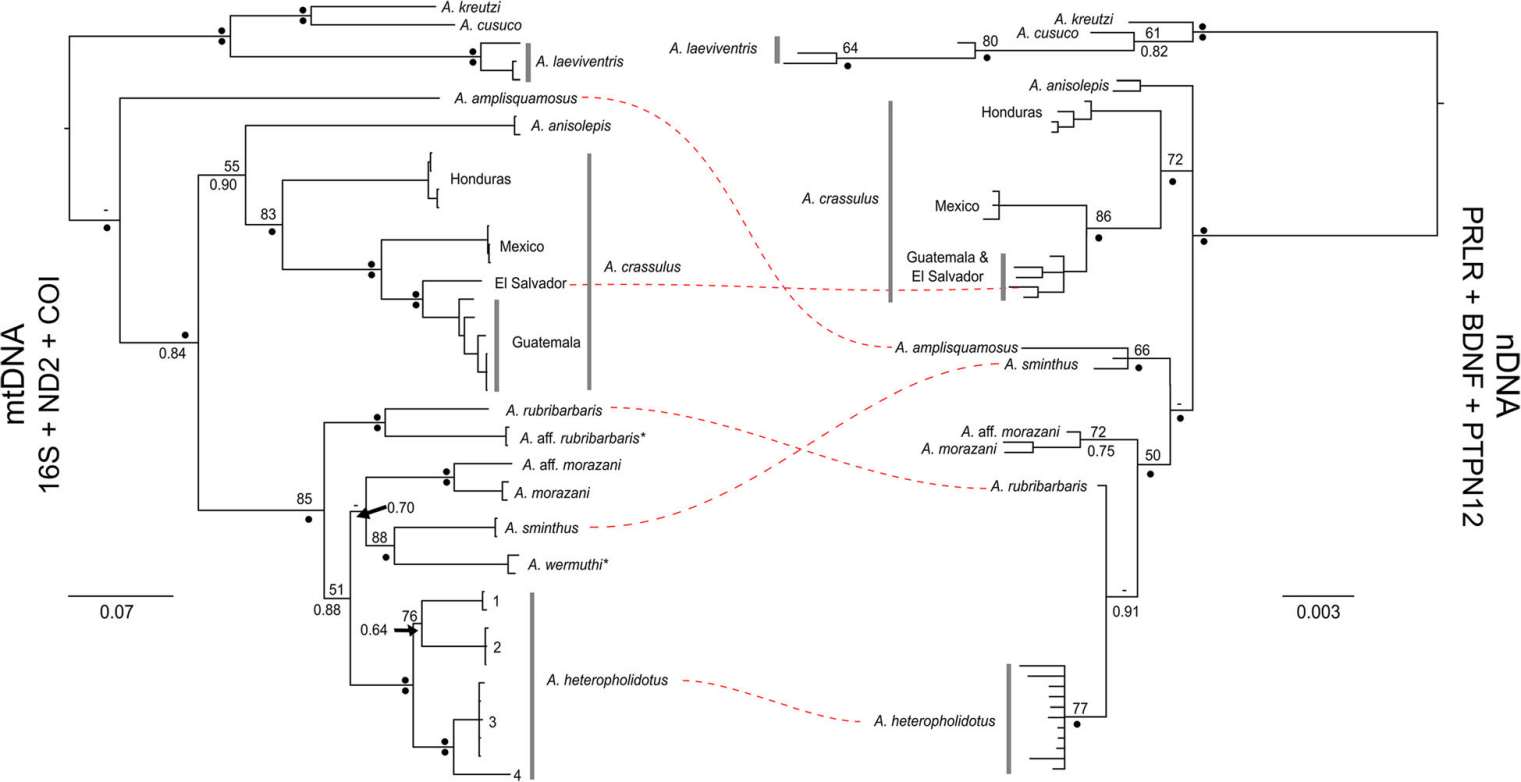
Comparison of Bayesian inference topologies for mtDNA (16S + ND2 + COI; left) and nDNA (PRLR + BDNF + PTPN12; right) datasets. Major differences in the recovery of lineages are marked with a red dashed line. Nodes are labelled with maximum likelihood bootstrap support (BS) on top, and Bayesian inference posterior probabilities (PP) on the bottom. Black circles indicate BS ≥ 95 or PP ≥ 0.95. BS < 50 and PP < 0.50 not shown. Asterisks indicate species not sequenced for nDNA loci

Analyses of mitochondrial-only and nuclear-only datasets were partially incongruent (Fig. 3). While both datasets recovered similar structure, the nuclear only dataset recovered a polytomy between the *A. anisolepis, A. crassulus* *sensu* *lato,* and the proposed *A. sminthus* clade. The placement of *Anolis amplisquamosus, A. rubribarbaris,* and *A. sminthus* were not congruent between analyses. Both datasets did recover multiple lineages assigned to *A. crassulus,* corresponding to geographic location, but the El Salvadoran population was nested within the Guatemalan samples in nuclear-only analyses. Interestingly, two samples previously identified as *A. crassulus* were recovered as a sister lineage to *A. rubribarbaris* (herein called *A.* aff. *rubribarbaris*). We were not able to include samples of *A.* aff. *rubribarbaris* or *A. wermuthi* in the remainder of the analyses due to a lack of viable tissue for sequencing. Four distinct lineages were recovered within *A. heteropholidotus* in mitochondrial-only analyses; this structure was not recovered in nuclear only analyses, which returned only a single lineage.

**Fig. 4:**
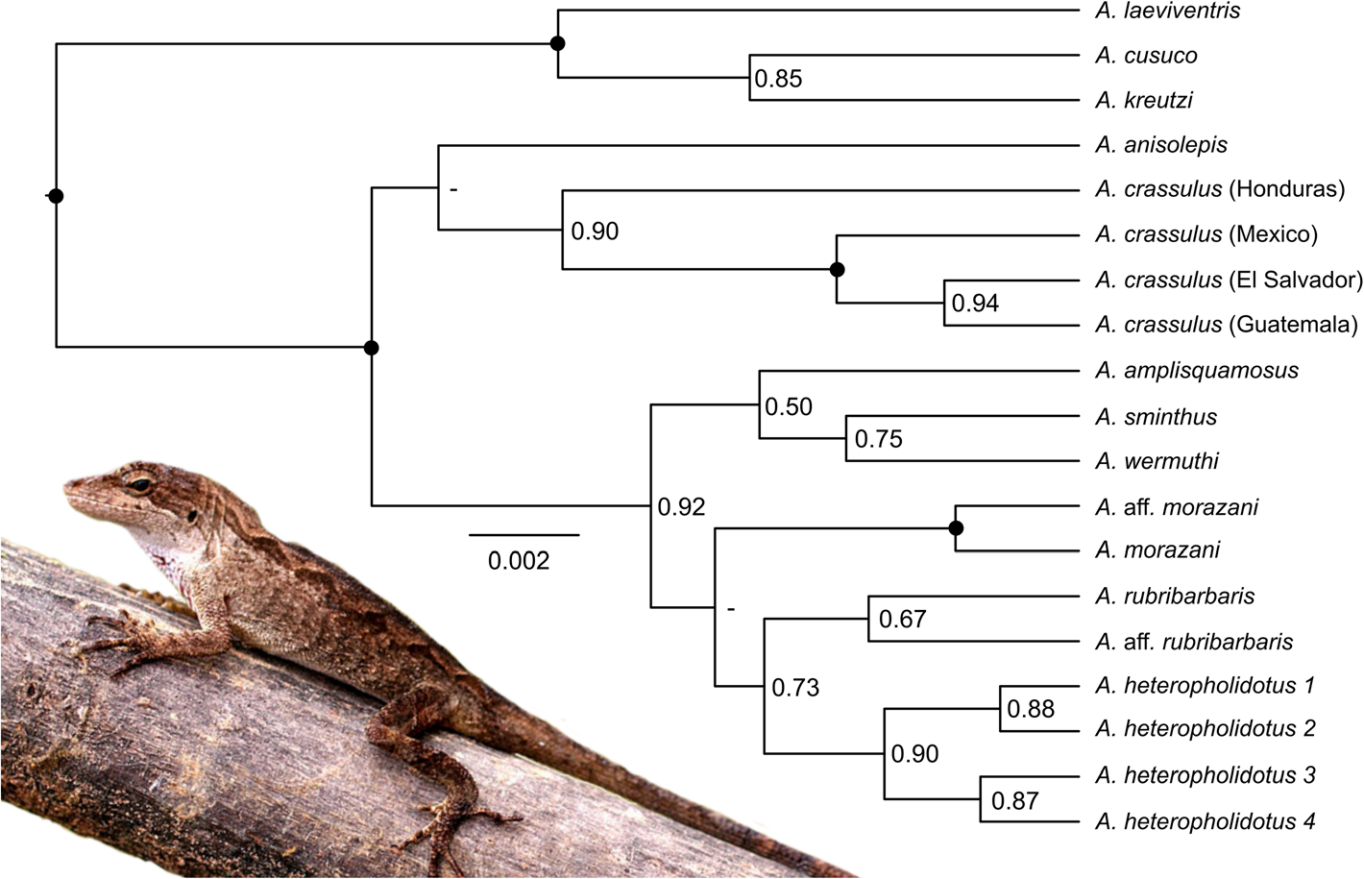
Species tree of the *Anolis crassulus* species subgroup based on the multispecies coalescent framework implemented in StarBEAST2. Black node circles indicate Bayesian posterior probabilities (PP) ≥ 0.95; PP < 0.50 not shown. Inset photo: *Anolis heteropholidotus* (2) from Dept. Ocotepeque, Honduras (JHT)

The species tree from the multispecies coalescent framework (Fig. 4) and the six-locus ML and BI topologies (Additional file 2: Figure S1) were largely congruent, supporting many of the relationships recovered from the other datasets analysed. Two clades within the *Anolis crassulus* subgroup were strongly supported (maximum likelihood bootstrap support (BS) = 100; Bayesian posterior probabilities (PP) = 1; species tree PP = 1). The substructure in *A. crassulus* was again recovered, with the Chortís population well-supported as basal to the other *A. crassulus* lineages (BS = 92; PP = 1/0.9), and the Mexican population strongly supported as distinct from the El Salvadoran/Guatemalan populations (BS = 100; PP = 1/1). As with earlier analyses, the placement of *A. amplisquamosus* differed between concatenated analyses (basal to the *A. sminthus* clade) and the species tree analyses (most closely related to *A. sminthus* and *A. wermuthi*), but was not well supported in either (BS = 43; PP = 0.52/0.50). *Anolis* aff. *morazani* was well-supported as a sister lineage to *A. morazani* (BS = 100; PP = 1/0.99). Four lineages were recovered within *A. heteropholidotus*, all well supported (BS = 100 for all splits except lineages 1 & 2, where BS = 83; PP > 0.99/0.87). The species tree analyses recovered *A. wermuthi* as sister to *A. sminthus,* and *A.* aff. *rubribarbaris* as sister to *A. rubribarbaris.* As only sequences of two mitochondrial loci were available for *A. wermuthi* and *A.* aff. *rubribarbaris,* they were not included in the concatenated, six-loci analyses*.* The addition of missing subgroup taxa (*Anolis haguei, A. muralla, A.* aff. *rubribarbaris,* and *A. wermuthi*) in the concatenated six-gene phylogeny may alleviate some of the incongruences apparent between the different analyses, and provide better resolution of the placement of some ambiguously placed taxa.

Within the *A. crassulus* lineages, average overall uncorrected pairwise (p) distances (of all six concatenated loci; Additional file 1: Appendix 5) ranged from 0.000–0.007, while between lineages, the average distances ranged from 0.032–0.071; between the Honduran population and the other populations of *A. crassulus,* these values ranged from 0.066–0.071. These inter-lineage distances are comparable to those between recognized taxa such as *A. rubribarbaris* and *A. morazani* (0.049), *A. rubribarbaris* and *A. anisolepis* (0.063), or *A. amplisquamosus* and *A. sminthus* (0.083). Honduran populations are deeply divergent compared to other populations across mtDNA (16S: 0.057–0.071; COI: 0.138–0.172; ND2: 0.138–0.169), and faster-evolving nNDA loci (PRLR: 0.016–0.022; PTPN12: 0.009–0.010), even retaining distinctiveness in the very-conserved BDNF (0.003–0.005).

### Divergence time estimation

**Fig. 5:**
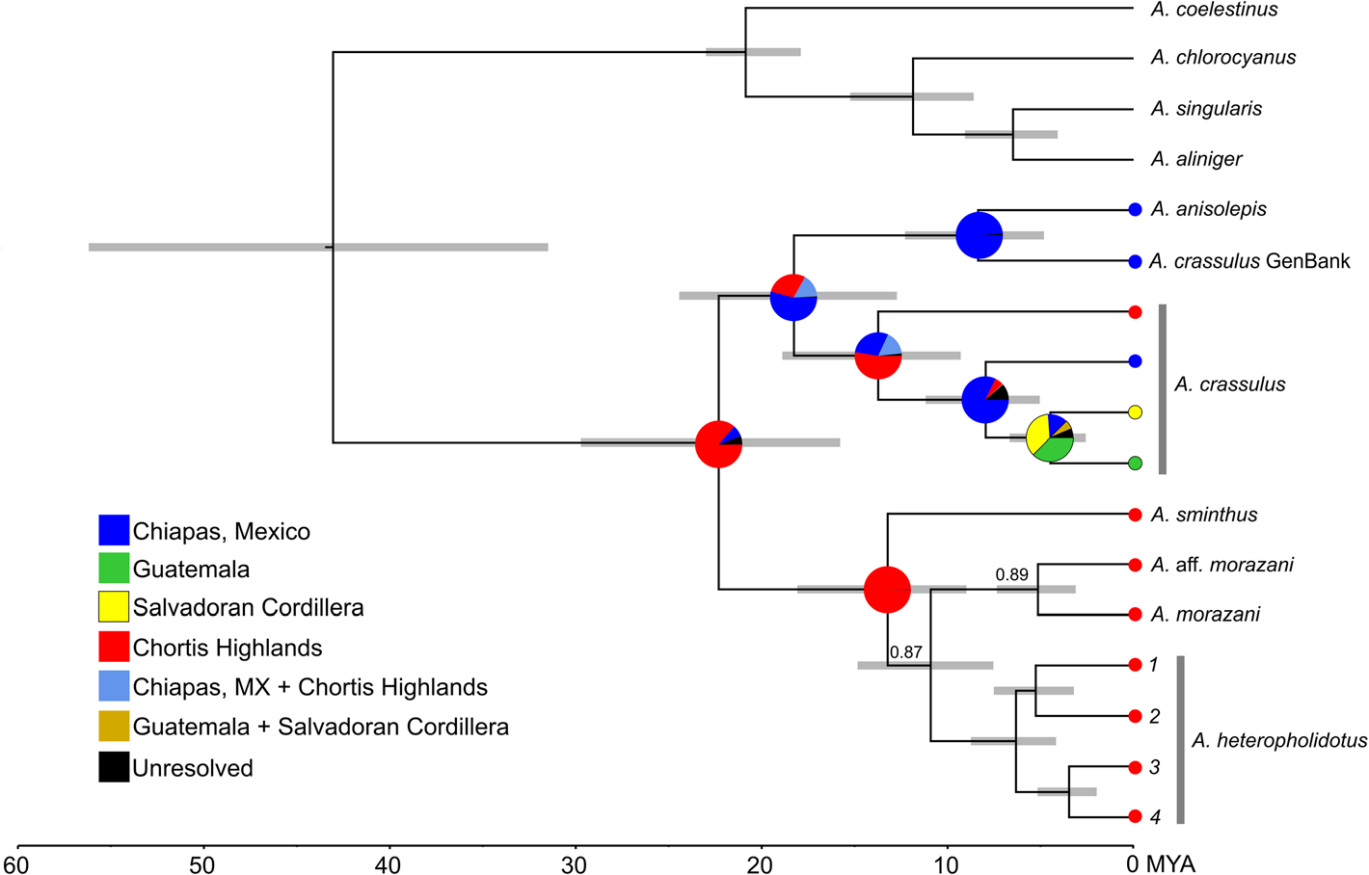
Chronogram of the *Anolis crassulus* subgroup resulting from divergence dating analysis in BEAST and ancestral area reconstruction with BBM in RASP. Dates are estimates based on the mtDNA locus ND2 with a calibration rate 0.765%/lineage/MY and a fossil calibration at the MRCA of the *A. chlorocyanus* group (17–23 MY). Node bars represent 95% highest posterior density intervals of the divergence time estimations. BEAST posterior probabilities are shown when <1. Pie charts node indicate probabilities for alternative ancestral range for MRCAs in the *A. crassulus* subgroup, corresponding to the color key; they are not shown within the *A. sminthus* clade as all ancestor distributions were reconstructed unambiguously as the Chortís Highlands (probability >0.99). Ingroup tips are colored according to extant distribution. Outgroup taxa were not included in the BBM analysis

For all divergence time estimations, the 95% highest posterior density (HPD) intervals (in millions of years) are presented in parenthesis after the estimated divergence time. Based on a rate of mutation of 0.765% per lineage per million years and a fossil calibration, the *Anolis crassulus* and *A. sminthus* clades were estimated to have diverged from each other approximately 22.3 MYA (15.8–29.7; Fig. 5). *Anolis anisolepis* and *A. crassulus* *sensu* *lato* were estimated to have diverged 18.3 MYA (12.7–24.4). *Anolis anisolepis* and the GenBank sample assigned to *A. crassulus* diverged 8.4 MYA (4.8–12.3). The Honduran lineage of *A. crassulus* was estimated to have diverged from the rest of *A. crassulus* *sensu* *lato* 13.7 MYA (9.3–18.9), and the lineage from Mexico diverged approximately 8.0 MYA (5.05–11.2). The El Salvadoran sample diverged from the Guatemalan *A. crassulus* approximately 4.5 MYA (2.6–6.7).

Within the *A. sminthus* clade, *A. sminthus* diverged from the other taxa approximately 13.2 MYA (9.0–18.1), and *A. morazani* *sensu* *lato* and *A. heteropholidotus* *sensu* *lato* diverged from each other 10.9 MYA (7.5–14.8). *Anolis* aff. *morazani* and *A. morazani* diverged from each other approximately 5.1 MYA (3.1–7.4). *Anolis heteropholidotus* lineages 1 and 2 were estimated to have diverged from lineages 3 and 4 approximately 6.3 MYA (4.2–8.8). Lineages 1 and 2 diverged from each other 5.3 MYA (3.2–7.5), and lineages 3 and 4 diverged from each other 3.5 MYA (2.0–5.2).

The divergence date estimates recovered here are similar to the larger squamate phylogeny of Zheng and Wiens [57] at two major nodes: the MRCA of the *Anolis chlorocyanus* outgroup and our ingroup taxa (43.1 compared to 43.6 MYA) and the *A. crassulus* and *A. sminthus* clades (22.3 MYA compared to 24.3 MYA). We recovered younger estimates within the *A. chlorocyanus* group: 20.9 MYA compared to 25.9 for the MRCA of *A. coelestinus* and the rest of the subgroup; 11.9 compared to 15.3 for the MRCA of *A. chlorocyanus, A. aliniger,* and *A. singularis*; and 6.5 compared to 8.8 for the MRCA of *A. aliniger* and *A. singularis.* Our estimates were comparable to Poe et al. [29], but not Nicholson et al. [18] (discussed below).

### Ancestral area reconstruction

The BBM analysis (Fig. 5) inferred the ancestral distributions of ingroup ancestors, though these results were not entirely unambiguous. The most recent common ancestor (MRCA) of the *Anolis crassulus* subgroup was inferred to be in the Chortís Highlands (probability = 86.4%). As expected, the MRCAs of the *Anolis sminthus* clade (all Chortís Highland endemics) were reconstructed as the Chortís Highlands (all nodes >99%). The ancestral areas of the *A. crassulus* clade, on the other hand, were reconstructed with less certainty. The MRCA of *A. anisolepis* and *A. crassulus* *sensu* *lato* was inferred to be in either the Chortís Highlands (54.2%) or in Chiapas, Mexico (28.9%), as was the MRCA of *A. crassulus* *sensu* *lato* (Chortís only: 52.5%; Mexico: 29.6%). The ancestor of the remaining *A. crassulus* lineages was likely found in Chiapas (82.3%), while the ancestor of El Salvadoran and Guatemalan populations was inferred to be in either Guatemala (37.4%) or the Salvadoran Cordillera (36.3%).

## Discussion

Our results indicate that significant evolutionary diversity within the *Anolis crassulus* subgroup has been overlooked. Congruent results from mtDNA, nDNA, concatenated, and species tree analyses revealed at least three deep genetic lineages presently assigned to *A. crassulus* *sensu* *lato,* including one lineage in the Chortís Block, *A. crassulus* *sensu stricto* in Guatemala and El Salvador, and a third lineage in Chiapas, Mexico. Importantly, the sample of *A. crassulus* from Chiapas, Mexico used in many recent anole phylogenies [18, 29, 30, 33] is not conspecific with *A. crassulus* *sensu stricto* *,* and instead represents a sister lineage to the Chiapan endemic *A. anisolepis.* Mitochondrial p-distances between Chortís Block *A. crassulus* and other populations of the species are notable in comparison to those of some recently described anoles, such as *A. purpuronectes* (ND2 *p* = 0.115 from its sister taxon *A. barkeri*; [33]), *A. kathydayae* (ND2 *p* = 0.125 from its congener *A. brooksi*; [61]), *A. mccraniei* and *A. wilsoni* (16S *p* = 0.032–0.044 from their congener *A. tropidonotus*; [62]), and *A. elcopeensis* (COI *p* ≥ 0.073 “from other included *Anolis* species”; [63]:4). Similar phylogeographic structure to *Anolis crassulus* *sensu* *lato* is apparent in the other taxa*.* Chortís Highland populations of the widespread pitviper *Cerrophidion godmani*, isolated from the Guatemalan populations by the same biogeographic boundaries that separate the lineages of *A. crassulus* *sensu* *lato*, were revealed to represent a distinct lineage and species (*C. wilsoni*; [64]). Similar structure is also apparent in montane mice of the *Peromyscus mexicanus* species complex, where numerous lineages are present throughout Chiapas, Guatemala, and western Honduras, corresponding to similar biogeographic breaks and highland localities [65].

Our BBM results suggest that the common ancestor of the *Anolis crassulus* subgroup was found in the Chortís Highlands, and the ancestor of non-Chortís *A. crassulus* lineages radiated north (Fig. 5). The BBM analysis suggested the possibility of a Mexico + Chortís Block distribution for the ancestor of *A. crassulus* and *A. anisolepis* lineages. While this seems unlikely given the modern placement of these land masses, it is plausible in light of currently accepted geologic hypotheses regarding the Chortís Block (as summarized in [66]). During the early Paleocene, the Chortís Block was located to the south of what is now mainland Mexico, disconnected from the rest of Central America [67]. Throughout the Paleocene, Eocene, and Oligocene epochs (between 65 and 20 MYA), the Chortís Block moved eastward along the southern margin of the North American Plate along the contemporary Pacific Coast of Mexico ([68]). Given the divergence time estimates and BBM results, we hypothesize the *A. crassulus* ancestor was distributed in the Chortís Block and subsequently invaded Mexico before further diversifying. It is also possible it was distributed in Mexico and the Chortís Block simultaneously during a period of connectivity between the two bodies, before the continuous eastward shift of the Chortís Block isolated the two areas. During the Miocene, intense volcanic activity in the region that is now southwestern Honduras, El Salvador, and southeastern Guatemala, likely isolated the populations [69, 70]. By the late Miocene, the Mexican lineage had subsequently invaded Guatemala and El Salvador, while the Chortís population remained isolated in the highlands of western Honduras. Rapid diversification also occurred in the Chortís Highlands within the *A. sminthus* clade, between 3.5–6.3 MYA.

Our results provide a clearer picture of the evolution of the *Anolis crassulus* subgroup than was available in either Nicholson et al. [18] or Poe et al. [29], due in large part to more complete molecular sampling of ingroup taxa. Our recovery of Nicholson et al. [18]’s *“A. sminthus”* (SMF 78830) as a divergent sister lineage to *A. morazani* *sensu stricto * makes sense in light of their phylogeny, which present the two as sister taxa. Our phylogenetic hypothesis is otherwise largely congruent with Nicholson et al. [18]. However, the timings of diversification presented by Nicholson et al. [18] are much older than those recovered here or by Poe et al. [29]. For example, Nicholson et al. [18] estimated the *A. crassulus* clade*/A. sminthus* clade divergence to have occurred at approximately 33 MYA, nearly 10 MY earlier than our estimate, and 17 MY earlier than the estimate of Poe et al. [29]. Their estimates for the *A. morazani/A.* aff. *morazani* and the *A. heteropholidotus/A. morazani* splits were between 7 and 10 MY earlier than our estimates. Our ancestral area reconstruction suggesting a Chortís origin and subsequent dispersal out for the *A. crassulus* subgroup is consistent with their results for the “*A. crassulus* clade” (consisting of more taxa than studied herein). However, they recovered a larger potential for a Mexican origin of *A. crassulus,* due to the fact their single sample of *A. crassulus* is from Chiapas, and not conspecific with *A. crassulus*.

Our phylogenetic hypothesis demonstrates numerous incongruences with those of Poe et al. [29], though our divergence time estimates were comparable. In an analysis that combined molecular-only, molecular + morphological, and morphological-only data into a single phylogeny, Poe et al. [29] presented a paraphyletic *A. crassulus* subgroup, recovering *A. crassulus, A. sminthus,* and *A. amplisquamosus* in a clade with *A. petersii,* distinct from any of the taxa they only scored for morphology. These were recovered in a different clade sister to *A. ortonii* and *A. sulcifrons*, along with some members of the *A. laeviventris* subgroup. These relationships were not well supported (posterior probabilities as low as 0.11), likely due to their use of morphology only for several taxa. Poe et al. [29] estimated the divergence between *A. crassulus* and *A. sminthus* clades to be 16 MYA, approximately 6.3 MY later than our estimate (22.3 MYA). Poe et al. [29] did not estimate other divergence times within our ingroup, but their estimates within the *A. chlorocyanus* group were similar to those recovered in this study, ranging from 2.5–3.9 MY later than our estimates. Given that we did not include the more conserved trailing tRNAs, and had no ingroup fossil calibrations, the dates we present for the *A. crassulus* subgroup may be slightly older than the actual divergence times in the group, and Poe et al. [29]’s estimate of the *A. crassulus/A. sminthus* clade split may be demonstrated to be more accurate. Until further calibrations within this subgroup can be implemented, however, our dates provide a general temporal context for the diversification of these taxa not previously sampled in any phylogeny.

The closest recorded localities between Chortís Highland *A. crassulus* (KU 209323) and Guatemalan *A. crassulus* (MVZ:HERP:160551) are approximately 115 km, separated by major rivers (including the Ríos Motagua, Ulúa, and Chamelecón) and an area of unsuitable habitat that dips below 150 m elevation (Fig. 1). The Río Motagua in particular is a well-known biogeographic vicariance, promoting diversification across lineages of numerous taxa, including snakes [12, 13], salamanders [16, 71], fishes [15], mice [14], insects [72], and squirrels [73]. The low- to mid-elevation areas in central Guatemala also correspond to diversification in snakes [12, 13], as well as scarabaeoids [74]. The closest samples of Chortís *A. crassulus* (KU 209323) and those from the Salvadoran Cordillera (KU 289793) are approximately 111 km apart, again separated by multiple rivers (including the Río Lempa) and an area dropping below 300 m elevation (Fig. 1).

While *Anolis crassulus* lineages are genetically distinct and have been isolated for millions of years, these allopatric lineages are highly conserved morphologically. Thorough morphological investigation into these lineages is underway, but initial examinations by the authors have failed to find consistent, discrete differences in morphological characters examined, including a lack of diagnosable differences in hemipenis or dewlap morphology. This is consistent with the results of past attempts to resolve these taxa (e.g. [20]) using morphology alone. Each lineage inhabits similar intermediate-to-high elevation Lower Montane Wet and Lower Montane Moist Forest formations [19]. This apparent lack of niche diversification has potentially limited selection that would lead to significant morphological differences, leaving these lineages with highly conserved morphologies. A thorough ecological study including niche modeling could find subtle but consistent differences between lineages, as much of the natural history of these animals remains unknown.

Based on the deep genetic differences between ingroup lineages, disjunct geographic distributions, the timing of diversification and potential distributions of the ancestor populations, and the apparent phenotypic and ecological stasis among populations, we hypothesize that the *Anolis crassulus* subgroup, and in particular *A. crassulus* *sensu* *lato,* represent a nonadaptive radiation of highland anoles. In contrast to adaptive radiations (such as those of the Caribbean anoles), where a single ancestor evolves to fill a variety of niche space, a nonadaptive radiation refers to “evolutionary diversification from a single clade, not accompanied by relevant niche differentiation” ([75]: 264). Nonadaptive radiations have been not been well documented in reptiles, but some studies have noted similar levels of diversity among cryptic lineages. Barley et al. [76, 77] found deep genetic diversification not accompanied by distinct morphological differentiation within cryptic lineages of the Asian skink genus *Eutropis,* which diverged between 1 and 10 MYA. A nonadaptive radiation has also been hypothesized as driving diversity in the South American lizard genus *Phymaturus* [78]. Within the *Anolis crassulus* subgroup, the majority of taxa and populations are allopatric, and have diverged within the last 15 MY. The subsequent genetic drift has left a series of genetically distinct, morphologically conserved, ecologically similar anoles across the Central American highlands.

It is clear that the *Anolis crassulus* subgroup is in need of a thorough taxonomic revision, as has been the case for some time [19–22]. Our results strongly support the validity of *Anolis anisolepis* and *A. heteropholidotus,* two taxa that previously have been questioned or inconsistently recognized. As for the rest of the subgroup, however, at this time we propose no other taxonomic changes until additional lines of evidence are fully examined. Continued morphological investigation into the Chortís populations of *A. crassulus* is underway, but given the distinct molecular substructure consistently recovered across all analyses, this population likely warrants species-level recognition. The unique Mexican lineages remain in need of further study. These populations are not as geographically isolated as Chortís populations relative to *A. crassulus* in Guatemala and El Salvador, suggesting additional modes of diversification within the group. Additional work is also underway to investigate populations assigned to *A.* aff. *morazani* and *A. heteropholidotus.*

## Conclusions

Based on analyses of the first multilocus dataset of the majority of the *Anolis crassulus* subgroup, we provide a new hypothesis for the evolutionary relationships, timing of diversification, and ancestral ranges of these anoles. Our results show clear molecular structure for numerous cryptic lineages within this group, and lay the groundwork for future investigation and potential taxonomic revisions. Additionally, our results support the validity of *A. anisolepis* and *A. heteropholidotus*. Based on these results, we hypothesize that this species complex represents a nonadaptive radiation of highland anoles. However, further investigation and studies of morphology, behavior, ecology, and diet, need to be undertaken to test this hypothesis.

By building upon this work with better taxonomic sampling and additional molecular, morphological, and ecological data, new questions about the diversity and biogeographic histories of mainland subgroups can be explored. Taken with other recent studies [8, 11, 17, 61], it is clear that the diversity in Mesoamerican anoles has been underestimated, and further investigation is needed to reveal the patterns of evolutionary diversification among these ubiquitous lizards.

## Additional files

Additional file 1: Appendix 1. Localities, samples, and GenBank Accession numbers for sequences used in this study. Taxa are identified by the genetic lineage they were recovered as in the phylogenetic analyses. **Appendix 2.** Primers and cycling parameters used in this study. **Appendix 3**. Additional ND2 samples and GenBank Accession numbers used in analyses of ND2-only data. **Appendix 4.** Models of nucleotide substitution as determined by PartitionFinder. **Appendix 5.** Uncorrected pairwise distances for each loci and overall, by recovered lineage. (DOCX 59 kb)

Additional file 2: Figure S1. Phylogenetic hypothesis of the *Anolis crassulus* species subgroup based on a concatenated datset of six loci analysed in RAxML and MrBayes. Nodes are labelled with maximum likelihood bootstrap support (BS; above), and Bayesian inference posterior probabilities (PP; below); Black circles indicate BS ≥ 95 or PP ≥ 0.95, and a single black circle on the node indicates both BS & PP ≥ 95 & 0.95, respectively. BS < 50 and PP < 0.50 not shown. Population names in red are those not recovered in an identical position to the StarBEAST2 species tree (Fig. 4). (TIFF 4374 kb)

## Abbreviations

16S: Mitochondrial 16S large subunit rRNA
AIC: Akaike information criterion
BBM: Bayesian Binary Markov-chain Monte Carlo
BDNF: Nuclear brain-derived neutrophic factor
BI: Bayesian inference
BOLD: Barcode of Life
bp: Basepairs
BS: Maximum likelihood bootstrap support
COI: Mitochondrial cytochrome oxidase subunit I
DMSO: Dimethyl sulfoxide
DNA: Deoxyribonucleic acid
dNTPs: Deoxynucleotides
EDTA: Ethylenediaminetetraacetic acid
ESS: Effective sample size
HPD: Highest posterior density
IUCN: International union for conservation of nature
JHT: Josiah H. Townsend
KU: University of Kansas Biodiversity Institute
LAB: Smithsonian Institution Laboratory of Analytical Biology
MCC: Maximum clade credibility
ML: Maximum likelihood
MRCA: Most recent common ancestor
mtDNA: Mitochondrial DNA
MVZ: Museum of Vertebrate Zoology, University of California Berkeley
MY: Million years
MYA: Million years ago
MZFC: Museo de Zoología “Alfonso L. Herrera” de la Facultad de Ciencias de la Universidad Nacional Autónoma de México
ND2: Mitochondrial NADH dehydrogenase subunit 2
nDNA: Nuclear DNA
PCR: Polymerase chain reaction
PP: Bayesian posterior probabilities
PRLR: Nuclear prolactin receptor
PTPN12: Nuclear protein tyrosine phosphatase non-receptor type 12
rRNA: Ribosomal ribonucleic acid
SED: Salt saturated NaCL-EDTA-DMSO solution
SMF: Senckenberg Forschungsinstitut und Naturmuseum
tRNA: Transfer RNA

## References

[1] Williams EE (1969). The ecology and colonization as seen in the zoogeography of anoline lizards on small islands. Q Rev Biol.

[2] Losos JB (2009). Lizards in an evolutionary tree: ecology and adaptive radiation of anoles.

[3] Losos JB, Jackman TR, Larson A, de Queiroz K, Rodríguez-Schettino L (1998). Contingency and determinism in replicated adaptive radiations of island lizards. Science.

[4] Beutell K, Losos JB (1999). Ecological morphology of Caribbean anoles. Herpetol Monogr.

[5] Losos JB, Miles DB (2002). Testing the hypothesis that a clade has adaptively radiated: iguanid lizard clades as a case study. Am Nat.

[6] Losos JB, Glor RE, Kolbe JJ, Nicholson K (2006). Adaptation, speciation, and convergence: a hierarchical analysis of adaptive radiation in Caribbean *Anolis* lizards. Ann Mo Bot Gard.

[7] Nicholson KE, Glor RE, Kolbe JJ, Larson A, Hedges SB, Losos JB (2005). Mainland colonization of island lizards. J Biogeogr.

[8] Prates I, Rodrigues MT, Melo-Sampaio PR, Carnaval AC (2015). Phylogenetic relationships of Amazonian anole lizards (*Dactyloa*): taxonomic implications, new insights about phenotypic evolution and the timing of diversification. Mol Phylogenet Evol.

[9] Prates I, Hernandez L, Samelo RR, Carnaval AC (2016). Molecular identification and geographic origin of an exotic anole lizard introduced to Brazil, with remarks on its natural history. S Am J Herpetol.

[10] Prates I, Rivera D, Rodrigues MT, Carnaval AC (2016). A mid-Pleistocene rainforest corridor enabled synchronous invasions of the Atlantic Forest by Amazonian anole lizards. Mol Ecol.

[11] Guarnizo CE, Werneck FP, Giugliano LG, Santos MG, Fenker J, Sousa L, D’Angiolella AB, dos Santos AR, Strüssman C, Rodrigues MT, Dorado-Rodrigues TF, Gamble T, Colli GR (2015). Cryptic lineages and diversification of an endemic anole lizard (Squamata, Dactyloidae) of the Cerrado hotspot. Mol Phylogenet Evol.

[12] Castoe TA, Daza JM, Smith EN, Sasa MM, Kuch U, Campbell JA, Chippindale PT, Parkinson CL (2009). Comparative phylogeography of pitvipers suggests a consensus of ancient Middle American highland biogeography. J Biogeogr.

[13] Daza JM, Smith EN, Páez VP, Parkinson CL (2009). Complex evolution in the Neotropics: the origin and diversification of the widespread genus *Leptodeira* (Serpentes: Colubridae). Mol Phylogenet Evol.

[14] Consuegra SGP, Vazquez-Dominguez E (2015). Mitochondrial diversification of *Peromyscus mexicanus* species group in Nuclear Central America: biogeographic and taxonomic implications. J Zool Syst Evol Res.

[15] Matamoros WA, McMahan CD, Chakrabarty P, Albert JS, Schaefer JF (2014). Derivation of the freshwater fish fauna of Central America revisited: Myer’s hypothesis in the twenty-first century. Cladistics.

[16] Rovito SM, Vásquez-Almazán CR, Papenfuss TJ, Parra-Olea G, Wake DB (2015). Biogeography and evolution of Central American cloud forest salamanders (Caudata: Plethodontidae: *Cryptotriton*), with the description of a new species. Zool J Linn Soc-Lond.

[17] Phillips JG, Deitloff J, Guyer C, Huetteman S, Nicholson KE (2015). Biogeography and evolution of a widespread Central American lizard species complex: *Norops humilis,* (Squamata: Dactyloidae). BMC Evol Biol.

[18] Nicholson KE, Guyer C, Phillips JG, Crother B, Parenti L (2017). Biogeographic origin of mainland *Norops* (Squamata: Dactyloidae). Assumptions inhibiting progress in comparative biology.

[19] McCranie JR, Köhler G (2015). The anoles (Reptilia: Squamata: Dactyloidae: *Anolis: Norops*) of Honduras. Systematics, distribution, and conservation. B Mus Comp Zool.

[20] Meyer JR, Wilson LD (1971). Taxonomic studies and notes on some Honduran amphibians and reptiles. B S Cal Acad Sci.

[21] Lieb CS (1981). Biochemical and karyological systematics of the Mexican lizards of the *Anolis gadovi* and *A. nebulosus* species groups (Reptilia: Iguanidae). Ph.D. Dissertation: University of California Los Angeles.

[22] McCranie JR, Wilson LD, Williams KL (1992). A new species of anole of the *Norops crassulus* group (Sauria: Polychridae) from northwestern Honduras. Caribb J Sci.

[23] Köhler G (2008). Reptiles of Central America.

[24] Townsend JH, Wilson LD (2009). New species of cloud forest *Anolis* (Squamata: Polychrotidae) of the *crassulus* group from Parque Nacional Montaña de Yoro, Honduras. Copeia.

[25] Lieb CS, Johnson JD, Webb RG, Flores-Villela OA (2001). Anole lizards of Mexico: a taxonomic overview. Mesoamerican herpetology: systematics, zoogeography, and conservation.

[26] Köhler G, McCranie JR, Wilson LD (1999). Two new species of anoles of the *Norops crassulus* group from Honduras (Reptilia: Sauria: Polychrotidae). Amphibia-Reptilia.

[27] Köhler G (1996). Notes on a collection of reptiles from El Salvador collected between 1951 and 1956. Senck Biol.

[28] Johnson JD, Mata-Silva V, Wilson LD (2015). A conservation reassessment of the Central American herpetofauna based on the EVS measure. Amphib Reptile Conse..

[29] Poe S, Nieto-Montes de Oca A, Torres-Carvajal O, de Queiroz K, Velasco JA, Truett B, Gray LN, Ryan MJ, Köhler G, Ayala-Varela F, Latella I (2017). A phylogenetic, biogeographic, and taxonomic study of all extant species of *Anolis* (Squamata; Iguanidae). Syst Biol.

[30] Nicholson KE, Crother BI, Guyer C, Savage JM (2012). It is time for a new classification of anoles (Squamata: Dacyloidae). Zootaxa.

[31] Mayer GC. Anolis sminthus. In: The IUCN red list of threatened species. IUCN. 2011. doi: 10.2305/IUCN.UK.2011-1.RLTS.T178291A7515435.en. Accessed 3 Jan 2017.

[32] Uetz P, Freed P, Hošek J (2016). The reptile database.

[33] Gray L, Meza-Lázaro R, Poe S, Nieto-Montes de Oca A (2016). A new species of semiaquatic *Anolis* (Squamata: Dacyloidae) from Oaxaca and Veracruz, Mexico. Herpetol J.

[34] Seutin G, White BN, Boag PT (1991). Preservation of avian blood and tissue samples for DNA analyses. Can J Zool.

[35] Williams ST (2007). 2007. Safe and legal shipment of tissue samples: does it affect DNA quality?. J Mollus Stud.

[36] Mulcahy DG, Macdonalad KS III, Brady SG, Meyer C, Barker KB, Coddington J. Greater than X kb: a quantitative assessment of preservation conditions on genomic DNA quality, and a proposed standard for genome-quality DNA. PeerJ. 2016; doi: 10.7287/peerj.preprints.2202v1. 10.7717/peerj.2528 PMC5068448 27761327

[37] Sabaj MH (2016). Standard symbolic codes for institutional resource collections in herpetology and ichthyology: an online reference. Version 6.5.

[38] Townsend TM, Alegre RE, Kelley ST, Wiens JJ, Reader TW (2008). Rapid development of multiple nuclear loci for phylogenetic analysis using genomic resources: an example from squamate reptiles. Mol Phylogenet Evol.

[39] Kearse M, Moir R, Wilson A, Stones-Havas S, Cheung M, Sturrock S, Buxton S, Cooper A, Markowitz S, Duran C, Thierer T (2012). Geneious basic: an integrated and extendable desktop software platform for the organization and analysis of sequence data. Bioinformatics.

[40] Kumar S, Stecher G, Tamura K (2016). MEGA: molecular evolutionary genetics analysis version .0 for bigger datasets. Mol Biol Evol.

[41] Thompson JD, Higgins DG, Gibson TJ (1994). ClustalW: improving the sensitivity of progressive multiple sequence alignment through sequence weighting, position-specific gap penalties and weight matrix choice. Nucleic Acids Res.

[42] Lanfear R, Calcott B, Ho SY, Guindon S (2016). ParitionFinder: combined selection of partitioning schemes and substitution models for phylogenetic analysis. Mol Biol Evol.

[43] Huelsenbeck JP, Ronquist F (2001). MRBAYES: Bayesian inference of phylogenetic trees. Bioinformatics.

[44] Ronquist F, Teslenko M, van der Mark P, Ayres DL, Darling A, Höhna S, Larget B, Liu L, Suchard MA, Huelsenbeck JP (2012). MrBayes 3.2: efficient Bayesian phylogenetic inference and model choice across a large model space. Syst Biol.

[45] Stamatakis A (2014). RAxML version 8: a tool for phylogenetic analysis and post-analysis of large phylogenies. Bioinformatics.

[46] Silvestro D, Michalak I (2012). raxmlGUI: a graphical front-end for RAxML. Org Divers Evol.

[47] Rambaut A. FigTree. http://tree.bio.ed.ac.uk/software/figtree. Accessed 1 May 2016.

[48] Inkscape. https://inkscape.org. Accessed 8 Dec 2016.

[49] Ogilvie HA, Bouckaert RR, Drummond AJ (2017). StarBEAST2 brings fasters species tree inference and accurate estimates of substitution rates. Mol Biol Evol.

[50] Bouckaert R, Heled J, Kühnert D, Vaughan T, Wu CH, Xie D, Suchard MA, Rambaut A, Drummond AJ (2014). BEAST 2: a software platform for Bayesian evolutionary analysis. PLoS Comput Biol.

[51] Leaché AD, Rannala B (2011). The accuracy of species tree estimation under simulation: a comparison of methods. Syst Biol.

[52] Maia-Carvalho B, Gonçalves H, Ferrand N, Martínez-Solano I (2014). Multilocus assessment of phylogenetic relationships in *Alytes* (Anura, Alytidae). Mol Phylogenet Evol.

[53] Rambaut A, Suchard MA, Xie D, Drummond AJ. Tracer v1.6. 2014. http://tree.bio.ed.ac.uk/software/tracer/. Accessed 1 Dec 2016.

[54] Drummond AJ, Suchard MA, Xie D, Rambaut A (2012). Bayesian phylogenetics with BEAUti and the BEAST 1.7. Mol Biol Evol.

[55] Macey JR, Schulte JA, Ananjeva NB, Larson A (1998). Phylogenetic relationships among agamid lizards of the *Laudakia caucasia* species group: testing hypotheses of biogeographic fragmentation and an area cladogram for the Iranian plateau. Mol Phylogenet Evol.

[56] Geneva AJ, Hilton J, Noll S, Glor RE (2015). Multilocus phylogenetic analyses of Hispaniolan and Bahamian trunk anoles (*distichus* species group). Mol Phylogenet Evol.

[57] Zheng Y, Wiens JJ (2016). Combining phylogenomic and supermatrix approaches, and a time-calibrated phylogeny for squamate reptiles (lizards and snakes) based on 52 genes and 4162 species. Mol Phylogenet Evol.

[58] de Queiroz K, Chu LR, Losos JB (1998). A second *Anolis* lizard in Dominican amber and the systematics and ecological morphology of Dominican amber anoles. Am Mus Novit.

[59] Ronquist F, Huelsenbeck JP (2003). MrBayes 3: Bayesian phylogenetic inference under mixed models. Bioinformatics.

[60] Yu Y, Harris AJ, Blair C, He XJ (2015). RASP (reconstruct ancestral state in phylogenies): a tool for historical biogeography. Mol Phylogenet Evol.

[61] Poe S, Ryan MJ (2017). Description of two new species similar to *Anolis insignis* (Squamata: Iguanidae) and resurrection of *Anolis (Diaphoranolis) brooksi*. Amphib Reptile Conse.

[62] Köhler G, Townsend JH, Petersen CBP (2016). A taxonomic revision of the *Norops tropidonotus* complex (Squamata, Dactyloidae), with the resurrection of *N. spilorhipis* (Álvarez del Toro and Smith, 1956) and the description of two new species. Mesoam Herpetol.

[63] Poe S, Scarpetta S, Schaad EW (2015). A new species of *Anolis* (Squamata: Iguanidae) from Panama. Amphib Reptile Conse.

[64] Jadin RC, Townsend JH, Castoe TA, Campbell JA (2012). Cryptic diversity in disjunct populations of Middle American Montane Pitvipers: a systematic reassessment of *Cerrophidion godmani*. Zool Scr.

[65] Pérez Consuegra SG, Vázquez-Domínguez E (2015). Mitochondrial diversification of the *Peromyscus mexicanus* species group in Nuclear Central America: biogeographic and taxonomic implications. J Zool Syst Evol Res.

[66] Townsend JH (2014). Characterizing the Chortís Block Biogeographic Province: geological, physiographic, and ecological associations and herpetofaunal diversity. Mesoam Herpetol.

[67] Rogers RD, Kárason H, van der Hilst RD (2002). Epeirogenic uplift above a detached slab in northern Central America. Geology.

[68] Rogers RD (2003). Jurassic-recent tectonic and stratigraphic history of the Chortís Block of Honduras and Nicaragua (northern Central America). Ph.D. dissertation: University of Texas at Austin.

[69] Jordan BR, Sigurdsson H, Carey SN. Ignimbrites in Central American and associated Caribbean Sea tephra: correlation and Petrogenesis. Saarbrücken: VDM Verlag.

[70] Gordan MB, Muehlberger WR (1994). Rotation of the Chortis block causes dextral slip on the Guayape fault. Tectonics.

[71] Rovito SM, Parra-Olea G, Vásquez-Almazán CR, Luna-Reyes R, Wake DB (2012). Deep divergences and extensive phylogeographic structure in a clade of lowland tropical salamanders. BMC Evol Biol.

[72] Sokolov IM, Kavanaugh DH (2014). The integripennis species group of *Geocharidius* Jeannel, 1963 (Carabidae, Bembidiini, Anillina) from Nuclear Central America: a taxonomic review with notes about biogeography and speciation. Zookeys.

[73] Villalobos F (2013). Tree squirrels: a key to understand the historic biogeography of Mesoamerica?. Mamm Biol.

[74] Schuster JC, Cano EB (2006). What can Scarabaeoidea contribute to the knowledge of the biogeography of Guatemala?. Coleopts Soc M.

[75] Gittenberger E (1991). What about non-adaptive radiation?. Biol J Linn Soc.

[76] Barley AJ, White J, Diesmos AC, Brown RM (2013). The challenge of species delimitation at the extremes: diversification without morphological change in Philippine sun skinks. Evolution.

[77] Barley AJ, Datta-Roy A, Karanth KP, Brown RM (2015). Sun skink diversification across the Indian–Southeast Asian biogeographical interface. J Biogeogr.

[78] Scolaro JA, Pincheira-Donoso D (2010). Lizards at the end of the world: two new species of *Phymaturus* of the *patagonicus* clade (Squamata, Liolaemidae) revealed in southern Patagonia of Argentina. Zootaxa.

