# Supplementary material for: Origins and biogeography of the Anolis crassulus subgroup (Squamata: Dactyloidae) in the highlands of Nuclear Central America
Source: BMC Evol Biol. 2017 Dec 21;17:267. doi: 10.1186/s12862-017-1115-8 (PMC5740896; doi:10.1186/s12862-017-1115-8)
Supplement: Supplementary file 1 — Localities, samples, and GenBank Accession numbers for sequences used in this study. Taxa are identified by the genetic lineage they were recovered as in the phylogenetic analyses. Appendix 2. Primers and cycling parameters used in this study. Appendix 3. Additional ND2 samples and GenBank Accession numbers used in analyses of ND2-only data. Appendix 4. Models of nucleotide substitution as determined by PartitionFinder. Appendix 5. Uncorrected pairwise distances for each loci and overall, by recovered lineage. (DOCX 59 kb) [file 12862_2017_1115_MOESM1_ESM.docx]

**Additional file 1:**

**Origins and biogeography of the *Anolis crassulus* subgroup (Squamata: Dactyloidae) in the highlands of Nuclear Central America**

Erich P. Hofmann^1,2*^ & Josiah H. Townsend^1,3^

* Correspondence: erich.p.hofmann@gmail.com

^1^ Department of Biology, Indiana University of Pennsylvania, Indiana, Pennsylvania, 15705-1081, USA

^2^ Present address: Department of Biological Sciences, Clemson University, Clemson, South Carolina, 29634, USA

^3^ email address: josiah.townsend@iup.edu

**Appendix 1**

Localities, samples, and GenBank Accession numbers for sequences used in this study. Taxa are identified by the genetic lineage they were recovered as in the phylogenetic analyses. ES: El Salvador; G: Guatemala; H: Honduras; M: Mexico; N: Nicaragua. An asterisk (*) following the GenBank ID in the ND2 column indicates the sample was used in the divergence dating and ancestral area reconstruction analyses. Sequences not generated as part of this study (and instead taken from GenBank) are marked with a dagger (†).

| **Taxon** | **Locality** | **Sample IDs**  **[Field #]** | **GenBank Accession Numbers** | | | | | |
| --- | --- | --- | --- | --- | --- | --- | --- | --- |
|  |  |  | *16S* | *ND2* | *COI* | *PRLR* | *BDNF* | *PTPN12* |
| *A. amplisquamosus* | H: Cortés: PN El Cusuco | UF 149642 | KU688052 | – | KU687941 | – | – | – |
|  | H: Cortés: PN El Cusuco | UF 149645 | KU688053 | – | KU687942 | MF094577 | MF094621 | – |
|  | H: Cortés: PN El Cusuco | UF 149647 | KU688051 | – | KU687940 | – | – | – |
|  | H: Cortés: PN El Cusuco | UF 149648 | KU688054 | – | KU687943 | – | – | – |
|  | H: Cortés: PN El Cusuco | USNM 578741 | KU688055 | – | KU687944 | – | – | – |
| *A. anisolepis* | M: Chiapas: Grutas de Rancho Nuevo | [GK5417] | MF094455 | – | MF094502 | MF094575 | MF094619 | MF094655 |
|  | M: Chiapas: Grutas de Rancho Nuevo | [GK5418] | MF094456 | MF094547* | MF094503 | MF094576 | MF094620 | – |
| *A. crassulus ES* | ES: Santa Ana: Finca El Milagro | KU 289793 | MF094477 | MF094564* | MF094524 | MF094607 | MF094642 | MF094670 |
|  | ES: Sonsonate: Cerro Verde | SMF 78094 | MF094497 | – | – | – | – | – |
|  | ES: Sonsonate: Cerro Verde | SMF 78098 | MF094498 | – | – | – | – | – |
|  | ES: Santa Ana: Volvan de Santa Ana | SMF 78099 | MF094499 | – | – | – | – | – |
| *A. crassulus G* | G: Guatemala: San Juan Sacatepéquez | SMF 82677 | MF094500 | – | MF094545 | – | – | – |
|  | G: Quetzaltenango: Fuentees Georginas | SMF 84431 | MF094501 | – | MF094546 | – | MF094654 | – |
|  | G: Jalapa: Cerro Miramundo | MVZ:HERP:263595 | MF094486 | MF094571 | MF094534 | MF094615 | MF094650 | MF094677 |
|  | G: El Quiche: NE of Laj Chimel | MVZ:HERP:265564 | MF094489 | – | MF094537 | – | – | – |
|  | G: El Quiche: NE of Laj Chimel | MVZ:HERP:265565 | MF094490 | MF094572* | MF094538 | MF094616 | MF094651 | MF094678 |
|  | G: El Quiche: NE of Laj Chimel | MVZ:HERP:265576 | MF094491 | MF094573 | MF094539 | MF094617 | MF094652 | MF094679 |
|  | G: El Quiche: NE of Laj Chimel | MVZ:HERP:265577 | MF094492 | MF094574 | MF094540 | MF094618 | MF094653 | MF094680 |
|  | G: El Quiche: NE of Laj Chimel | MVZ:HERP:265578 | MF094493 | – | MF094541 | – | – | – |
|  | G: El Quiche: NE of Laj Chimel | MVZ:HERP:265579 | MF094494 | – | MF094542 | – | – | – |
|  | G: El Quiche: NE of Laj Chimel | MVZ:HERP:265580 | MF094495 | – | MF094543 | – | – | – |
|  | G: Chimaltenango: Cerro Balamjuyu | MVZ:HERP:270039 | MF094496 | – | MF094544 | – | – | – |
| *A. crassulus H* | H: La Paz: Near Guajiquiro | UF 166186 | KU688065 | – | KU687954 | – | – | – |
|  | H: La Paz: Near Guajiquiro | UF 166188 | KU688066 | – | KU687955 | – | – | – |
|  | H: Intibucá: Zacate Blanco | UF 166190 | KU688060 | – | KU687949 | – | – | – |
|  | H: Intibucá: Zacate Blanco | UF 166191 | KU688061 | – | KU687950 | – | – | – |
|  | H: Intibucá: towers above Buenos Aires | [JHT2891] | KU688062 | – | KU687951 | – | – | – |
|  | H: Intibucá: towers above Buenos Aires | [JHT2892] | KU688063 | – | KU687952 | – | – | – |
|  | H: Intibucá: towers above Buenos Aires | [JHT2894] | MF094466 | – | MF094513 | – | – | – |
|  | H: Intibucá: towers above Buenos Aires | [JHT2895] | KU688067 | – | KU687956 | – | – | – |
|  | H: Intibucá: RVS Mixcure: El Rodeo | [JHT3768] | MF094468 | MF094560* | MF094515 | MF094598 | MF094634 | MF094661 |
|  | H: Intibucá: RVS Mixcure: El Rodeo | [JHT3770] | MF094469 | MF094561 | MF094516 | MF094599 | MF094635 | MF094662 |
|  | H: Intibucá: RB Opalaca: Rio Agua Negra | [JHT3822] | MF094471 | – | MF094518 | MF094601 | MF094637 | MF094664 |
|  | H: Intibucá: RB Opalaca: Rio Agua Negra | [JHT3888] | MF094472 | MF094562 | MF094519 | MF094602 | MF094638 | MF094665 |
| *A. crassulus M* | M: Chiapas: Cerro Boqueron | MVZ:HERP:256801 | MF094480 | – | MF094528 | – | – | – |
|  | M: Chiapas: Cerro Boqueron | MVZ:HERP:256802 | MF094481 | MF094568* | MF094529 | MF094612 | MF094647 | MF094674 |
|  | M: Chiapas: Cerro Boqueron | MVZ:HERP:256803 | MF094482 | MF094569 | MF094530 | MF094613 | MF094648 | MF094675 |
|  | M: Chiapas: Cerro Boqueron | MVZ:HERP:256804 | MF094483 | MF094570 | MF094531 | MF094614 | MF094649 | MF094676 |
|  | M: Chiapas: Cerro Boqueron | MVZ:HERP:256805 | MF094484 | – | MF094532 | – | – | – |
|  | M: Chiapas: Cerro Boqueron | MVZ:HERP:256806 | MF094485 | – | MF094533 | – | – | – |
| *A. crassulus* GenBank† | M: Chiapas: “Mpio. Unión Juárez”^1^ “2 km E Chiquihuite”^2^ | MZFC 6458 | – | KT724758* | – | – | – | – |
| *A. heteropholidotus 1* | H: Ocotepeque: Cerro Montecristo | [MMF005] | KU688076 | – | MF094527 | MF094610 | MF094645 | MF094673 |
|  | H: Ocotepeque: Cerro Montecristo | [MMF006] | KU688078 | MF094567* | KU687966 | MF094611 | MF094646 | – |
| *A. heteropholidotus 2* | ES: Chalatenango: Cerro El Pital | KU 291250 | MF094478 | MF094565 | MF094525 | MF094608 | MF094643 | MF094671 |
|  | ES: Chalatenango: Cerro El Pital | KU 291251 | MF094479 | MF094566* | MF094526 | MF094609 | MF094644 | MF094672 |
|  | H: Ocotepeque: RB El Güisayote | UF 166194 | KU688082 | – | KU687970 | – | – | – |
|  | H: Ocotepeque: RB El Güisayote | UF 166195 | KU688085 | – | KU687973 | MF094589 | – | – |
|  | H: Ocotepeque: RB El Güisayote | UF 166196 | KU688090 | MF094555 | KU687978 | MF094590 | MF094630 | – |
|  | H: Ocotepeque: RB El Güisayote | UF 166197 | MF094461 | – | MF094508 | – | – | – |
|  | H: Ocotepeque: RB El Güisayote | UF 166198 | KU688074 | – | KU687963 | MF094591 | – | – |
|  | H: Ocotepeque: RB El Güisayote | UF 166199 | KU688084 | – | KU687972 | – | – | – |
|  | H: Ocotepeque: RB El Güisayote | UF 166200 | MF094462 | – | MF094509 | – | – | – |
|  | H: Ocotepeque: RB El Güisayote | UF 166201 | KU688077 | – | KU687965 | – | – | – |
|  | H: Ocotepeque: RB El Güisayote | UF 166202 | MF094463 | – | MF094510 | – | – | – |
|  | H: Ocotepeque: RB El Güisayote | UF 166203 | MF094464 | – | MF094511 | – | – | – |
|  | H: Ocotepeque: RB El Güisayote | UF 166204 | KU688075 | – | KU687964 | – | – | – |
|  | H: Ocotepeque: RB El Güisayote | UF 166207 | KU688086 | – | KU687974 | – | – | – |
|  | H: Ocotepeque: RB El Güisayote | UF 166208 | MF094465 | – | MF094512 | – | – | – |
|  | H: Ocotepeque: RB El Güisayote | UF 166209 | KU688087 | – | KU687975 | – | – | – |
|  | H: Ocotepeque: El Portillo | MVZ:HERP:263596 | MF094487 | – | MF094535 | – | – | – |
|  | H: Ocotopeque: RB Güisayote | MVZ:HERP:263869 | MF094488 | – | MF094536 | – | – | – |
| *A. heteropholidotus 3* | H: Intibucá: Cerro San Pedro | UF 166279 | KU688073 | – | KU687962 | – | – | – |
|  | H: Intibucá: Cerro San Pedro | UF 166280 | KU688080 | – | KU687968 | – | – | – |
|  | H: Intibucá: Cerro San Pedro | UF 166281 | MF094458 | – | MF094505 | – | – | – |
|  | H: Intibucá: Cerro San Pedro | UF 166283 | KU688083 | – | KU687971 | – | – | – |
|  | H: Intibucá: Cerro San Pedro | UF 166284 | KU688088 | – | KU687976 | – | – | – |
|  | H: Intibucá: Cerro San Pedro | UF 166285 | KU688089 | – | KU687977 | – | – | – |
|  | H: Intibucá: Cerro San Pedro | UF 166287 | MF094459 | – | MF094506 | – | – | – |
|  | H: Intibucá: Cerro San Pedro | UF 166288 | MF094460 | – | MF094507 | – | – | – |
|  | H: Intibucá: towers above San Pedro La Loma | [JHT2885] | KU688081 | – | KU687969 | – | – | – |
|  | H: Intibucá: towers above Buenos Aires | [JHT2893] | KU688079 | – | KU687967 | – | – | – |
|  | H: Intibucá: San Pedro La Loma | [JHT3818] | MF094470 | – | MF094517 | MF094600 | MF094636 | MF094663 |
|  | H: Intibucá: RB Opalaca: Rio Agua Negra | [JHT3891] | MF094473 | – | MF094520 | MF094603 | MF094639 | MF094666 |
|  | H: Intibucá: RB Opalaca: Rio Agua Negra | [JHT3892] | MF094474 | – | MF094521 | MF094604 | MF094640 | MF094667 |
|  | H: Intibucá: RB Opalaca: Rio Agua Negra | [JHT3893] | MF094475 | – | MF094522 | MF094605 | MF094641 | MF094668 |
|  | H: Intibucá: RB Opalaca: Rio Agua Negra | [JHT3903] | MF094476 | MF094563* | MF094523 | MF094606 | – | MF094669 |
| *A. heteropholidotus 4* | H: Lempira: PN Celaque | [JHT3639] | MF094467 | MF094559* | MF094514 | MF094597 | MF094633 | – |
| *A. morazani* | H: Francisco Morazán: PN Montaña de Yoro | UF 151792 | KU688119 | MF094549 | KU688007 | MF094579 | MF094623 | MF094657 |
|  | H: Francisco Morazán: PN Montaña de Yoro | UF 151757 | KU688121 | MF094550* | KU688009 | MF094580 | MF094624 | – |
|  | H: Francisco Morazán: PN Montaña de Yoro | UF 151784 | KU688124 | – | KU688012 | – | – | – |
|  | H: Francisco Morazán: PN Montaña de Yoro | UF 151791 | KU688115 | – | KU688003 | – | – | – |
|  | H: Francisco Morazán: PN Montaña de Yoro | UF 151758 | KU688126 | – | KU688014 | – | – | – |
|  | H: Francisco Morazán: PN Montaña de Yoro | UF 151767 | KU688125 | – | KU688013 | – | – | – |
|  | H: Francisco Morazán: PN Montaña de Yoro | UF 151765 | KU688120 | – | KU688008 | – | – | – |
|  | H: Francisco Morazán: PN Montaña de Yoro | UF 166241 | KU688123 | – | KU688011 | MF094592 | – | – |
|  | H: Francisco Morazán: PN Montaña de Yoro | UF 166242 | KU688117 | – | KU688005 | MF094593 | – | – |
| *A.* aff. *morazani* | H: Olancho: PN Sierra de Agalta | USNM 578764 | KU688122 | – | KU688010 | – | – | – |
|  | H: Olancho: PN Sierra de Agalta | USNM 578765 | KU688116 | MF094558 | KU688004 | MF094596 | – | – |
|  | H: Olancho: PN Sierra de Agalta | USNM 578766 | KU688118 | – | KU688006 | – | – | – |
| † | H: Olancho: “Pico La Picucha” ^1^ | SMF 78830 | – | AY909779* | – | – | – | – |
| *A. rubribarbaris 1* | H: Santa Bárbara: PN Santa Barbara | UF 152661 | KU688135 | – | KU688023 | – | – | – |
|  | H: Santa Bárbara: PN Santa Barbara | UF 152660 | KU688136 | – | KU688024 | MF094588 | MF094629 | MF094660 |
|  | H: Santa Bárbara: PN Santa Barbara | UF 152662 | KU688137 | – | KU688025 | – | – | – |
|  | H: Santa Bárbara: PN Santa Barbara | USNM 578761 | KU688134 | – | KU688022 | – | – | – |
| *A.* aff. *rubribarbaris* | H: La Paz: Near Guajiquiro | UF 166189 | KU688059 | – | KU687948 | – | – | – |
|  | H: La Paz: Near Guajiquiro | [JHT2876] | KU688064 | – | KU687953 | – | – | – |
| *A. sminthus* | H: Comayagua: PN Montana de Comayagua | UF 166266 | KU688140 | – | KU688028 | – | – | – |
|  | H: Comayagua: PN Montana de Comayagua | UF 166267 | KU688141 | – | KU688029 | MF094583 | – | – |
|  | H: Comayagua: PN Montana de Comayagua | UF 166268 | KU688138 | – | KU688026 | MF094584 | – | – |
|  | H: Comayagua: PN Montana de Comayagua | UF 166269 | KU688139 | MF094553* | KU688027 | MF094585 | MF094627 | MF094658 |
|  | H: Comayagua: PN Montana de Comayagua | UF 166270 | KU688142 | MF094554 | KU688030 | MF094586 | MF094628 | MF094659 |
|  | H: Comayagua: PN Montana de Comayagua | UF 166272 | KU688145 | – | KU688033 | MF094587 | – | – |
|  | H: Comayagua: PN Montana de Comayagua | UF 166273 | KU688146 | – | KU688034 | – | – | – |
|  | H: Comayagua: PN Montana de Comayagua | UF 166274 | KU688144 | – | KU688032 | – | – | – |
|  | H: Comayagua: PN Montana de Comayagua | UF 166275 | KU688143 | – | KU688031 | – | – | – |
|  | H: Comayagua: PN Montana de Comayagua | UF 166276 | MF094457 | – | MF094504 | – | – | – |
|  | H: Comayagua: PN Montana de Comayagua | UF 166277 | KU688147 | – | KU688035 | – | – | – |
| *A. wermuthi* | N: Jinotega: Reserva Natural Cerro Kilambé | [N562] | KU688149 | – | KU688037 | – | – | – |
|  | N: Jinotega: Reserva Natural Cerro Kilambé | [N956] | KU688148 | – | KU688036 | – | – | – |
| *A. cusuco* | H: Cortés: Parque Nacional Cusuco | USNM 578744 | KU688068 | MF094556 | KU687957 | MF094594 | MF094631 | – |
| *A. kreutzi* | H: Atlántida: RVS Texiguat, La Liberacion | USNM 578819 | KU688097 | MF094557 | KU687985 | MF094595 | MF094632 | – |
| *A. laeviventris* | H: Francisco Morazán: PN La Tigra | [JHT2000] | KU688110 | MF094548 | KU687998 | MF094578 | MF094622 | MF094656 |
|  | H: Francisco Morazán: Finca la Alondra | [JHT2229] | KU688108 | MF094551 | KU687996 | MF094581 | MF094625 | – |
|  | N: Matagalpa: Reserva Selva Negra | [JHT2284] | KU688104 | MF094552 | KU687992 | MF094582 | MF094626 | – |
| *A. aliniger* † | – | – | – | AF055959* | – | – | – | – |
| *A. chlorocyanus*† | – | [GLOR1104] | – | EF531574* | – | – | – | – |
| *A. coelestinus*† | – | – | – | AF055958* | – | – | – | – |
| *A. singularis*† | – | – | – | AY296202* | – | – | – | – |

^1^ Nicholson [1]
^2^ Gray et al. [2]

**Appendix 2**

Primers and cycling parameters used in this study.

| **Gene** | **Primer Name** | **Primer Sequence (5′–3′)** | **Reference** | **Cycling Parameters** |
| --- | --- | --- | --- | --- |
| *16S* | 16Sar | CGCCTGTTTATCAAAAACAT | [3] | 94°C 3 min; 35x [94°C 45 s, 50°C 45 s, 72°C 45 s]; 72°C 5 min |
|  | 16Sbr | CCGGTCTGAACTCAGATCACGT | [3] |  |
| *ND2* | LVT_5617 | AAAGTGYTTGAGTTGCATTCA | [4] | 94°C 2 min; 30x [94°C 35 s, 52°C 35 s, 72°C 90 s]; 72°C 10 min |
|  | LVT_Metf.6 | AAGCTATTGGGCCCATACC | [4] |  |
| *COI* | dgLCO-1490 | GGTCAACAAATCATAAAGAYATYGG | [5] | 94°C 90 s; 37x [94°C 40 s, 45°C 40 s, 72°C 40 s]; 72°C 6 min |
|  | dgHCO-2198 | TAAACTTCAGGGTGACCAAARAAYCA | [5] |  |
| *PRLR* | PRLR_f1 | GACARYGARGACCAGCAACTRATGCC | [6] | 95°C 90 s; 10x [95°C 35 s, 63°C (-0.5°C/cycle) 35 s, 72°C 60 s]; 10x [95°C 35 s, 58°C 35s, 72°C 1 min]; 15x [94°C 35s, 52°C 35s, 72°C 60 s]; 72°C 10 min |
|  | PRLR_r3 | GACYTTGTGRACTTCYACRTAATCCAT | [6] |  |
| *BDNF* | BDNF_rep_F | GACCATCCTTTTCCTKACTATGGTTATTTCATACTT | [6] | 94°C 3 min; 10x [94°C 30 s, 55°C 30 s, 72°C 90 s]; 25x [94°C 30 s, 50°C 30s, 72°C 90 s]; 72°C 5 min |
|  | BDNF_rep_R | CTATCTTCCCCTTTTAATGGTCAGTGTACAAAC | [6] |  |
| *PTPN12* | PTPN12_F1 | AGTTGCCTTGTWGAAGGRGATGC | [6] | 94°C 5 min; 38x [94°C 45 s, 58°C 45 s, 72°C 60 s]; 72°C 10 min |
|  | PTPN12_R6 | CTRGCAATKGACATYGGYAATAC | [6] |  |

**Appendix 3**

Additional ND2 samples and GenBank Accession numbers used in analyses of the ND2-only dataset. All samples originally from Nicholson [1] or Nicholson et al. [7] except where noted.

| **Species** | **Sample ID (Field or Museum)** | **GenBank Accession Number** |
| --- | --- | --- |
| *Anolis allisoni*^1^ | - | AY296151 |
| *A. altae* | MVCFC 14383 | AY909735 |
| *A. annectens* | CIEZAH 1159 | AY909736 |
| *A. aquaticus* | JMS 63 | AY909738 |
| *A. auratus* | CIEZAH 1163 | AY909740 |
| *A. bicaorum* | LDW 12476 | AY909741 |
| *A. bitectus* | KU 218372 | AY909743 |
| *A. boulengerianus*^2^ | MFO 191 | AY909762 |
| *A. capito* | JMS 59 | AY909744 |
| *A. casildae* | JMS 214 | AY909745 |
| *A. cooki* | KDQ 1029 | AY909747 |
| *A. crassulus*^3^ | MZFC 6458 (FDQ 1529) | KT724758 |
| *A. cupeyalensis* | SBH 191155 | AY909749 |
| *A. cupreus* | JMS 67 | AY909750 |
| *A. cyanopleurus* | SBH 190289 | AY909751 |
| *A. frenatus* | JMS 192 | AY909752 |
| *A. guazuma* | KDQ 1640 | AY909754 |
| *A. intermedius*^4^ | MVCFC 14224 | AY909755 |
| *A. kemptoni* | JMS 35 | AY909770 |
| *A. laeviventris* | MVCFC 12252 | AY909756 |
| *A. lionotus* | JMS 208 | AY909757 |
| *A. lividus* | KDQ 1137 | AY909758 |
| *A. loveridgei* | USNM 10683 | AY909759 |
| *A. meridionalis* | LF 166692 | AY909760 |
| *A. nebuloides* | LCM 105 | AY909763 |
| *A. nubilus* | KDQ 1077 | AY909764 |
| *A. onca* | CIEZAH 1154 | AY909765 |
| *A. oporinus* | SBH 191409 | AY909766 |
| *A. oscelloscapularis* | SMF 79078 | AY909767 |
| *A. oxylophus* | LDW 11853 | AY909768 |
| *A. pachypus* | JMS 175 | AY909769 |
| *A. poecilopus* | JMS 194 | AY909771 |
| *A. polylepis* | JMS 46 | AY909772 |
| *A. purpurgularis* | USNM 508433 | AY909774 |
| *A. quercorum* | MZFC 7832 | AY909775 |
| *A. rejectus* | KDQ 2090 | AY909761 |
| *A. rubiginosus*^5^ | ENS 10040 | AY909773 |
| *A. rubribarbus*^6^ | GLOR 2719 | AY909789 |
| *A. sabanus* | JBL 942 | AY909776 |
| *A. schwartzi* | USNM 321927 | AY909777 |
| *A. sericeus* | LACM 7069 | AY909778 |
| *A. sminthus* | SMF 78830 (LDW 11414) | AY909779 |
| *A. trinitatis* | - | AY909781 |
| *A. tropidogaster* | JMS 203 | AY909782 |
| *A. tropidonotus*^7^ | SMF 78831 | AY909783 |
| *A. uniformis* | - | AY909784 |
| *A. utilensis* | LDW 12480 | AY909785 |
| *A. zeus* | LDW 13076 | AY909786 |

^1^ Harmon et al. [8]

^2^ Sequence labelled *A. isthmicus* (synonymized by Nieto-Montes de Oca et al. [9])

^3^ Gray et al. [2]. Note: this is the same specimen as was used in Nicholson [1] and Nicholson et al. [10]. However, this is a longer, cleaner sequence, including ND2.

^4^ Some experts consider *A. intermedius* to be a synonym of *A. laeviventris* (see McCranie et al. [11]). However, it is still listed as a valid taxon by Uetz et al. [12], and the results of our ND2 phylogeny (Fig. 2) recover it as a lineage distinct from *A. laeviventris*.

^5^ Sequence labelled *A. polyrachis* (synonymized by Nieto-Montes de Oca et al. [13])

^6^ Note that *A. rubribarbus* Barbour & Ramsden and *A. rubribarbaris* (Köhler, McCranie, & Wilson) are two different species. *Anolis rubribarbus* is endemic to Cuba [14] and not closely related to the *A. crassulus* subgroup of Nuclear Central America.

^7^ Referred to *Anolis* (*Norops*) *mccraniei* by Köhler et al. [15]

**Appendix 4**

Models of nucleotide substitution as determined by PartitionFinder v1.1.1.

| **Gene** | **Codon Position** | **ML & BI Models: RAxML/MrBayes** | **Multispecies Coalescent Model: StarBEAST2** |
| --- | --- | --- | --- |
| 16S | *gene* | GTR | GTR+I+G |
|  | 1^st^ | GTR+I+G (GTR+I+G) |  |
| ND2 (ND2 only) | 2^nd^ | HKY+G  (HKY+I+G) | TrN+I+G |
|  | 3^rd^ | GTR+G  (GTR+G) |  |
|  | 1^st^ | SYM+I+G |  |
| COI | 2^nd^ | HKY+I+G | TrN+I+G |
|  | 3^rd^ | GTR+G |  |
|  | 1^st^ | GTR+G |  |
| PRLR | 2^nd^ | HKY+G | HKY+I |
|  | 3^rd^ | HKY+G |  |
|  | 1^st^ | HKY |  |
| BDNF | 2^nd^ | K80+I+G | HKY+I |
|  | 3^rd^ | HKY |  |
|  | 1^st^ | HKY+I |  |
| PTPN12 | 2^nd^ | HKY+G | GTR+G |
|  | 3^rd^ | GTR+G |  |
| ND2  Divergence Dating with  *A. chlorocyanus* subgroup | *gene* | TrN+I+G |  |

**Appendix 5**

Uncorrected pairwise distances for each loci and overall, by recovered lineage. Intraspecific distances are shaded.

| **16S** | *amplisq-uamosus* | *anisolepis* | *crassulus* | | | | *heteropholidotus* | | | | *mora-zani* | aff*.  mora-zani* | *rubri-barbaris* | aff*. rubri-barbaris* | *smin-thus* | *werm-uthi* |
| --- | --- | --- | --- | --- | --- | --- | --- | --- | --- | --- | --- | --- | --- | --- | --- | --- |
|  |  |  | El Salvador | Guatemala | Honduras | Mexico | 1 | 2 | 3 | 4 |  |  |  |  |  |  |
| *amplis-quamosus* | 0.003 |  |  |  |  |  |  |  |  |  |  |  |  |  |  |  |
| *anisolepis* | 0.100 | 0.005 |  |  |  |  |  |  |  |  |  |  |  |  |  |  |
| *crassulus (El Salvador)* | 0.078 | 0.071 | 0.001 |  |  |  |  |  |  |  |  |  |  |  |  |  |
| *crassulus (Guatemala)* | 0.083 | 0.073 | 0.008 | 0.001 |  |  |  |  |  |  |  |  |  |  |  |  |
| *crassulus (Honduras)* | 0.096 | 0.082 | 0.057 | 0.064 | 0.005 |  |  |  |  |  |  |  |  |  |  |  |
| *crassulus (Mexico)* | 0.085 | 0.085 | 0.026 | 0.024 | 0.071 | 0.000 |  |  |  |  |  |  |  |  |  |  |
| *heteroph-olidotus 1* | 0.102 | 0.089 | 0.053 | 0.057 | 0.076 | 0.059 | 0.000 |  |  |  |  |  |  |  |  |  |
| *heteroph-olidotus 2* | 0.098 | 0.090 | 0.054 | 0.057 | 0.075 | 0.068 | 0.017 | 0.003 |  |  |  |  |  |  |  |  |
| *heteroph-olidotus 3* | 0.108 | 0.092 | 0.060 | 0.064 | 0.077 | 0.067 | 0.024 | 0.021 | 0.000 |  |  |  |  |  |  |  |
| *heteroph-olidotus 4* | 0.101 | 0.089 | 0.060 | 0.064 | 0.079 | 0.070 | 0.030 | 0.024 | 0.012 | - |  |  |  |  |  |  |
| *morazani* | 0.106 | 0.100 | 0.064 | 0.068 | 0.082 | 0.066 | 0.035 | 0.034 | 0.036 | 0.038 | 0.004 |  |  |  |  |  |
| aff*. morazani* | 0.105 | 0.103 | 0.061 | 0.065 | 0.089 | 0.072 | 0.041 | 0.036 | 0.047 | 0.048 | 0.033 | 0.002 |  |  |  |  |
| *rubri-barbarbis* | 0.100 | 0.091 | 0.049 | 0.054 | 0.072 | 0.065 | 0.031 | 0.021 | 0.041 | 0.043 | 0.040 | 0.030 | 0.001 |  |  |  |
| aff*. rubri-barbaris* | 0.107 | 0.095 | 0.059 | 0.064 | 0.080 | 0.066 | 0.034 | 0.028 | 0.034 | 0.042 | 0.032 | 0.038 | 0.026 | 0.004 |  |  |
| *sminthus* | 0.110 | 0.094 | 0.063 | 0.068 | 0.089 | 0.074 | 0.039 | 0.034 | 0.049 | 0.047 | 0.049 | 0.053 | 0.044 | 0.050 | 0.006 |  |
| *wermuthi* | 0.099 | 0.094 | 0.060 | 0.065 | 0.080 | 0.076 | 0.036 | 0.032 | 0.047 | 0.048 | 0.038 | 0.034 | 0.030 | 0.044 | 0.048 | 0.005 |

| **ND2** | *anisolepis* | *crassulus* | | | | | *heteropholidotus* | | | | *morazani* | aff*. morazani* | *sminthus* | *sminthus* GenBank *(SMF 78830)* |
| --- | --- | --- | --- | --- | --- | --- | --- | --- | --- | --- | --- | --- | --- | --- |
|  |  | El Salvador | Guatemala | Honduras | Mexico | GenBank(MZFC 6458) | 1 | 2 | 3 | *4* |  |  |  |  |
| *anisolepis* | - |  |  |  |  |  |  |  |  |  |  |  |  |  |
| *crassulus (El Salvador)* | 0.190 | - |  |  |  |  |  |  |  |  |  |  |  |  |
| *crassulus (Guatemala)* | 0.196 | 0.084 | 0.020 |  |  |  |  |  |  |  |  |  |  |  |
| *crassulus (Honduras)* | 0.192 | 0.169 | 0.172 | 0.008 |  |  |  |  |  |  |  |  |  |  |
| *crassulus (Mexico)* | 0.196 | 0.119 | 0.119 | 0.166 | 0.007 |  |  |  |  |  |  |  |  |  |
| *GenBank crassulus (MZFC 6458)* | 0.122 | 0.194 | 0.186 | 0.199 | 0.190 | - |  |  |  |  |  |  |  |  |
| *heteropholidotus 1* | 0.214 | 0.208 | 0.211 | 0.224 | 0.201 | 0.202 | - |  |  |  |  |  |  |  |
| *heteropholidotus 2* | 0.223 | 0.226 | 0.221 | 0.211 | 0.204 | 0.213 | 0.097 | 0.002 |  |  |  |  |  |  |
| *heteropholidotus 3* | 0.201 | 0.199 | 0.204 | 0.208 | 0.192 | 0.193 | 0.091 | 0.093 | - |  |  |  |  |  |
| *heteropholidotus 4* | 0.203 | 0.202 | 0.203 | 0.212 | 0.194 | 0.197 | 0.101 | 0.112 | 0.064 | - |  |  |  |  |
| *morazani* | 0.219 | 0.203 | 0.214 | 0.213 | 0.213 | 0.210 | 0.145 | 0.153 | 0.131 | 0.149 | 0.011 |  |  |  |
| aff*. morazani* | 0.214 | 0.200 | 0.207 | 0.213 | 0.209 | 0.202 | 0.150 | 0.161 | 0.146 | 0.159 | 0.072 | - |  |  |
| *sminthus* | 0.211 | 0.221 | 0.227 | 0.208 | 0.211 | 0.204 | 0.159 | 0.147 | 0.143 | 0.157 | 0.164 | 0.170 | 0.002 |  |
| *sminthus* GenBank *(SMF 78830)* | 0.228 | 0.210 | 0.213 | 0.229 | 0.218 | 0.215 | 0.155 | 0.173 | 0.172 | 0.182 | 0.101 | 0.023 | 0.188 | - |

| **COI** | *amplisq-uamosus* | *anisolepis* | *crassulus* | | | | *heteropholidotus* | | | | *mora-zani* | aff*.  mora-zani* | *rubri-barbaris* | *aff. rubri-barbaris* | *smin-thus* | *werm-uthi* |
| --- | --- | --- | --- | --- | --- | --- | --- | --- | --- | --- | --- | --- | --- | --- | --- | --- |
|  |  |  | El Salvador | Guatemala | Honduras | Mexico | 1 | 2 | 3 | 4 |  |  |  |  |  |  |
| *amplis-quamosus* | 0.006 |  |  |  |  |  |  |  |  |  |  |  |  |  |  |  |
| *anisolepis* | 0.209 | 0.000 |  |  |  |  |  |  |  |  |  |  |  |  |  |  |
| *crassulus (El Salvador)* | 0.180 | 0.168 | - |  |  |  |  |  |  |  |  |  |  |  |  |  |
| *crassulus (Guatemala)* | 0.183 | 0.156 | 0.054 | 0.010 |  |  |  |  |  |  |  |  |  |  |  |  |
| *crassulus (Honduras)* | 0.181 | 0.149 | 0.141 | 0.138 | 0.007 |  |  |  |  |  |  |  |  |  |  |  |
| *crassulus (Mexico)* | 0.182 | 0.153 | 0.091 | 0.088 | 0.138 | 0.001 |  |  |  |  |  |  |  |  |  |  |
| *heteroph-olidotus 1* | 0.193 | 0.189 | 0.165 | 0.168 | 0.171 | 0.176 | 0.002 |  |  |  |  |  |  |  |  |  |
| *heteroph-olidotus 2* | 0.181 | 0.190 | 0.161 | 0.166 | 0.174 | 0.175 | 0.065 | 0.001 |  |  |  |  |  |  |  |  |
| *heteroph-olidotus 3* | 0.179 | 0.185 | 0.165 | 0.168 | 0.170 | 0.172 | 0.066 | 0.065 | 0.002 |  |  |  |  |  |  |  |
| *heteroph-olidotus 4* | 0.187 | 0.177 | 0.163 | 0.162 | 0.163 | 0.167 | 0.073 | 0.068 | 0.044 | - |  |  |  |  |  |  |
| *morazani* | 0.187 | 0.202 | 0.170 | 0.184 | 0.179 | 0.181 | 0.133 | 0.140 | 0.146 | 0.136 | 0.004 |  |  |  |  |  |
| aff*. morazani* | 0.179 | 0.188 | 0.169 | 0.180 | 0.173 | 0.190 | 0.118 | 0.137 | 0.132 | 0.127 | 0.071 | 0.015 |  |  |  |  |
| *rubri-barbarbis* | 0.166 | 0.175 | 0.174 | 0.176 | 0.179 | 0.184 | 0.131 | 0.132 | 0.127 | 0.130 | 0.154 | 0.136 | 0.003 |  |  |  |
| *aff. rubri-barbaris* | 0.192 | 0.186 | 0.164 | 0.171 | 0.178 | 0.163 | 0.141 | 0.143 | 0.137 | 0.137 | 0.144 | 0.137 | 0.123 | 0.002 |  |  |
| *sminthus* | 0.183 | 0.180 | 0.165 | 0.170 | 0.149 | 0.169 | 0.109 | 0.128 | 0.122 | 0.124 | 0.110 | 0.110 | 0.146 | 0.140 | 0.010 |  |
| *wermuthi* | 0.200 | 0.196 | 0.170 | 0.168 | 0.168 | 0.177 | 0.127 | 0.136 | 0.125 | 0.131 | 0.125 | 0.124 | 0.151 | 0.150 | 0.098 | 0.010 |

| **PRLR** | *ampli-squamosus* | *anisolepis* | *crassulus* | | | | *heteropholidotus* | | | | *morazani* | aff*. morazani* | *rubri-barbaris* | *sminthus* |
| --- | --- | --- | --- | --- | --- | --- | --- | --- | --- | --- | --- | --- | --- | --- |
|  |  |  | El Salvador | Guatemala | Honduras | Mexico | 1 | 2 | 3 | 4 |  |  |  |  |
| *amplisquamosus* | - |  |  |  |  |  |  |  |  |  |  |  |  |  |
| *anisolepis* | 0.023 | 0.004 |  |  |  |  |  |  |  |  |  |  |  |  |
| *crassulus (El Salvador)* | 0.024 | 0.016 | - |  |  |  |  |  |  |  |  |  |  |  |
| *crassulus (Guatemala)* | 0.019 | 0.013 | 0.009 | 0.008 |  |  |  |  |  |  |  |  |  |  |
| *crassulus (Honduras)* | 0.022 | 0.015 | 0.020 | 0.016 | 0.002 |  |  |  |  |  |  |  |  |  |
| *crassulus (Mexico)* | 0.023 | 0.024 | 0.021 | 0.019 | 0.022 | 0.000 |  |  |  |  |  |  |  |  |
| *heteropholidotus 1* | 0.017 | 0.019 | 0.016 | 0.012 | 0.021 | 0.023 | 0.005 |  |  |  |  |  |  |  |
| *heteropholidotus 2* | 0.016 | 0.017 | 0.014 | 0.010 | 0.020 | 0.022 | 0.003 | 0.002 |  |  |  |  |  |  |
| *heteropholidotus 3* | 0.015 | 0.019 | 0.014 | 0.011 | 0.021 | 0.022 | 0.004 | 0.002 | 0.002 |  |  |  |  |  |
| *heteropholidotus 4* | 0.017 | 0.019 | 0.015 | 0.012 | 0.023 | 0.022 | 0.004 | 0.003 | 0.004 | - |  |  |  |  |
| *morazani* | 0.023 | 0.015 | 0.012 | 0.009 | 0.017 | 0.019 | 0.014 | 0.012 | 0.012 | 0.013 | 0.003 |  |  |  |
| aff*. morazani* | 0.020 | 0.012 | 0.009 | 0.006 | 0.015 | 0.016 | 0.011 | 0.010 | 0.010 | 0.011 | 0.003 | - |  |  |
| *rubribarbaris* | 0.020 | 0.012 | 0.009 | 0.006 | 0.015 | 0.017 | 0.009 | 0.008 | 0.008 | 0.009 | 0.003 | 0.000 | - |  |
| *sminthus* | 0.010 | 0.011 | 0.013 | 0.008 | 0.015 | 0.021 | 0.010 | 0.008 | 0.008 | 0.009 | 0.011 | 0.008 | 0.008 | 0.000 |

| **BDNF** | *amplisquamosus* | *anisolepis* | *crassulus* | | | | *heteropholidotus* | | | | *morazani* | *rubribarbaris* | *sminthus* |
| --- | --- | --- | --- | --- | --- | --- | --- | --- | --- | --- | --- | --- | --- |
|  |  |  | El Salvador | Guatemala | Honduras | Mexico | 1 | 2 | 3 | 4 |  |  |  |
| *amplisquamosus* | - |  |  |  |  |  |  |  |  |  |  |  |  |
| *anisolepis* | 0.004 | 0.000 |  |  |  |  |  |  |  |  |  |  |  |
| *crassulus (El Salvador)* | 0.010 | 0.008 | - |  |  |  |  |  |  |  |  |  |  |
| *crassulus (Guatemala)* | 0.010 | 0.008 | 0.003 | 0.003 |  |  |  |  |  |  |  |  |  |
| *crassulus (Honduras)* | 0.004 | 0.003 | 0.005 | 0.005 | 0.000 |  |  |  |  |  |  |  |  |
| *crassulus (Mexico)* | 0.008 | 0.007 | 0.002 | 0.002 | 0.003 | 0.000 |  |  |  |  |  |  |  |
| *heteropholidotus 1* | 0.004 | 0.003 | 0.005 | 0.005 | 0.000 | 0.003 | 0.000 |  |  |  |  |  |  |
| *heteropholidotus 2* | 0.004 | 0.003 | 0.005 | 0.005 | 0.000 | 0.003 | 0.000 | 0.000 |  |  |  |  |  |
| *heteropholidotus 3* | 0.005 | 0.004 | 0.005 | 0.005 | 0.000 | 0.004 | 0.000 | 0.000 | 0.001 |  |  |  |  |
| *heteropholidotus 4* | 0.004 | 0.003 | 0.005 | 0.005 | 0.000 | 0.003 | 0.000 | 0.000 | 0.000 | - |  |  |  |
| *morazani* | 0.008 | 0.007 | 0.011 | 0.012 | 0.007 | 0.010 | 0.007 | 0.007 | 0.007 | 0.007 | 0.000 |  |  |
| *rubribarbaris* | 0.004 | 0.003 | 0.005 | 0.005 | 0.000 | 0.003 | 0.000 | 0.000 | 0.000 | 0.000 | 0.007 | - |  |
| *sminthus* | 0.000 | 0.003 | 0.008 | 0.008 | 0.003 | 0.007 | 0.003 | 0.003 | 0.004 | 0.003 | 0.007 | 0.003 | 0.000 |

| **PTPN12** | *anisolepis* | *crassulus* | | | | *heteropholidotus* | | | *morazani* | *rubribarbaris* | *sminthus* |
| --- | --- | --- | --- | --- | --- | --- | --- | --- | --- | --- | --- |
|  |  | El Salvador | Guatemala | Honduras | Mexico | 1 | 2 | 3 |  |  |  |
| *anisolepis* | - |  |  |  |  |  |  |  |  |  |  |
| *crassulus (El Salvador)* | 0.012 | - |  |  |  |  |  |  |  |  |  |
| *crassulus (Guatemala)* | 0.011 | 0.001 | 0.001 |  |  |  |  |  |  |  |  |
| *crassulus (Honduras)* | 0.009 | 0.010 | 0.009 | 0.001 |  |  |  |  |  |  |  |
| *crassulus (Mexico)* | 0.011 | 0.005 | 0.004 | 0.010 | 0.001 |  |  |  |  |  |  |
| *heteropholidotus 1* | 0.010 | 0.014 | 0.013 | 0.012 | 0.013 | - |  |  |  |  |  |
| *heteropholidotus 2* | 0.010 | 0.013 | 0.013 | 0.011 | 0.013 | 0.001 | 0.000 |  |  |  |  |
| *heteropholidotus 3* | 0.007 | 0.012 | 0.011 | 0.011 | 0.012 | 0.003 | 0.002 | 0.000 |  |  |  |
| *heteropholidotus 4* | 0.012 | 0.018 | 0.017 | 0.014 | 0.017 | 0.011 | 0.011 | 0.008 | - |  |  |
| *morazani* | 0.006 | 0.012 | 0.012 | 0.010 | 0.012 | 0.004 | 0.003 | 0.000 | 0.007 | - |  |
| *rubribarbaris* | 0.008 | 0.014 | 0.014 | 0.012 | 0.014 | 0.009 | 0.009 | 0.005 | 0.010 | 0.005 | 0.004 |

| **FULL DATASET** | *ampli-squamosus* | *anisolepis* | *crassulus* | | | | *heteropholidotus* | | | | *morazani* | aff*. morazani* | *rubri-barbaris* | *sminthus* |
| --- | --- | --- | --- | --- | --- | --- | --- | --- | --- | --- | --- | --- | --- | --- |
|  |  |  | El Salvador | Guatemala | Honduras | Mexico | 1 | 2 | 3 | 4 |  |  |  |  |
| *amplisquamosus* | - |  |  |  |  |  |  |  |  |  |  |  |  |  |
| *anisolepis* | 0.093 | 0.002 |  |  |  |  |  |  |  |  |  |  |  |  |
| *crassulus (El Salvador)* | 0.082 | 0.082 | - |  |  |  |  |  |  |  |  |  |  |  |
| *crassulus (Guatemala)* | 0.080 | 0.076 | 0.032 | 0.007 |  |  |  |  |  |  |  |  |  |  |
| *crassulus (Honduras)* | 0.083 | 0.071 | 0.071 | 0.066 | 0.002 |  |  |  |  |  |  |  |  |  |
| *crassulus (Mexico)* | 0.082 | 0.080 | 0.052 | 0.048 | 0.069 | 0.001 |  |  |  |  |  |  |  |  |
| *heteropholidotus 1* | 0.087 | 0.085 | 0.080 | 0.079 | 0.079 | 0.082 | 0.001 |  |  |  |  |  |  |  |
| *heteropholidotus 2* | 0.081 | 0.094 | 0.100 | 0.096 | 0.090 | 0.098 | 0.032 | 0.001 |  |  |  |  |  |  |
| *heteropholidotus 3* | 0.089 | 0.076 | 0.065 | 0.062 | 0.064 | 0.065 | 0.026 | 0.026 | 0.001 |  |  |  |  |  |
| *heteropholidotus 4* | 0.085 | 0.094 | 0.108 | 0.106 | 0.103 | 0.109 | 0.039 | 0.052 | 0.020 | - |  |  |  |  |
| *morazani* | 0.087 | 0.100 | 0.102 | 0.104 | 0.099 | 0.108 | 0.062 | 0.078 | 0.056 | 0.084 | 0.005 |  |  |  |
| aff*. morazani* | 0.112 | 0.131 | 0.134 | 0.137 | 0.134 | 0.145 | 0.083 | 0.105 | 0.080 | 0.105 | 0.052 | - |  |  |
| *rubribarbaris* | 0.078 | 0.063 | 0.052 | 0.049 | 0.053 | 0.054 | 0.040 | 0.036 | 0.036 | 0.049 | 0.049 | 0.065 | - |  |
| *sminthus* | 0.083 | 0.088 | 0.097 | 0.095 | 0.084 | 0.096 | 0.055 | 0.069 | 0.046 | 0.086 | 0.078 | 0.105 | 0.041 | 0.002 |

**Appendix Literature Cited**

1. Nicholson KE. Phylogenetic analysis and a test of the current infrageneric classification of *Norops* (beta *Anolis*). Herpetol Monogr. 2002:16:93–120.

2. Gray L, Meza-Lázaro R, Poe S, Nieto-Montes de Oca A. A new species of semiaquatic *Anolis* (Squamata: Dacyloidae) from Oaxaca and Veracruz, Mexico. Herpetol J. 2016;26:253–62.

3. Palumbi SR, Martin A, Romano S, McMillan WO, Stice L, Grabowski G. The Simple Fool’s Guide to PCR. 1^st^ ed. Honolulu: University of Hawaii: 1991

4. Rodríguez-Robles JA, Jezkova T, García MA. Evolutionary relationships and historical biogeography of *Anolis desechensis* and *Anolis monensis,* two lizards endemic to small islands in the eastern Caribbean Sea. J Biogeog. 2007:34:1546–58.

5. Meyer CP. Molecular systematics of cowries (Gastropoda: Cypraeidae) and diversification patterns in the tropics. Biol J Linn Soc. 2003:79:401–59.

6. Townsend TM, Alegre RA, Kelley ST, Wiens JJ, Reeder TW. Rapid development of multiple nuclear loci for phylogenetic analysis using genomic resources: An example from squamate reptiles. Mol Phylogenet Evol. 2008:47:129–42.

7. Nicholson KE, Glor RE, Kolbe JJ, Larson A, Hedges SB, Losos JB. Mainland colonization of island lizards. J Biogeogr. 2005;32:929–38.

8. Harmon LJ, Schulte II JA, Larson A, Losos JB. Tempo and mode of evolutionary radiation in iguanian lizards. Science. 2003:301:961–4.

9. Nieto-Montes de Oca A, Köhler G, Feria-Ortiz M. *Anolis boulengerianus* Thominot, 1887, a senior synonym of *Anolis isthmicus* Fitch, 1978 (Squamata: Dactyloidae). Zootaxa. 2014:3794:125–33.

10. Nicholson KE, Crother BI, Guyer C, Savage JM. It is time for a new classification of anoles (Squamata: Dacyloidae). Zootaxa. 2012;3477:1–108.

11. McCranie JR, Köhler G, Wilson LD. Two new species of anoles from northwestern Honduras related to *Norops laeviventris* (Wiegmann 1834). Senck Biol. 2000:80:213–23.

12. Uetz P, Freed P, Hošek J (eds.). The Reptile Database. <http://www.reptile-database.org>. Accessed 24 July 2017.

13. Nieto-Montes de Oca A, Poe S, Scarpetta S, Gray L, Lieb CS. Synonyms for some species of Mexican anoles (Squamata: Dactyloidae). Zootaxa. 2013:3637:484–92.

14. Rodríguez Schettino L, Mancina CA, González VR. Reptiles of Cuba: checklist and geographic distributions. Smithsonian Herp Inf Serv. 2013; 144:1–96.

15. Köhler G, Townsend JH, Petersen CBP. A taxonomic revision of the *Norops tropidonotus* complex (Squamata, Dactyloidae), with the resurrection of *N. spilorhipis* (Álvarez del Toro and Smith, 1956) and the description of two new species. Mesoam Herpetol. 2016;3:8–41.
