# Supplementary figures and images for: Origins and biogeography of the Anolis crassulus subgroup (Squamata: Dactyloidae) in the highlands of Nuclear Central America
Source: BMC Evol Biol. 2017 Dec 21;17:267. doi: 10.1186/s12862-017-1115-8 (PMC5740896; doi:10.1186/s12862-017-1115-8)

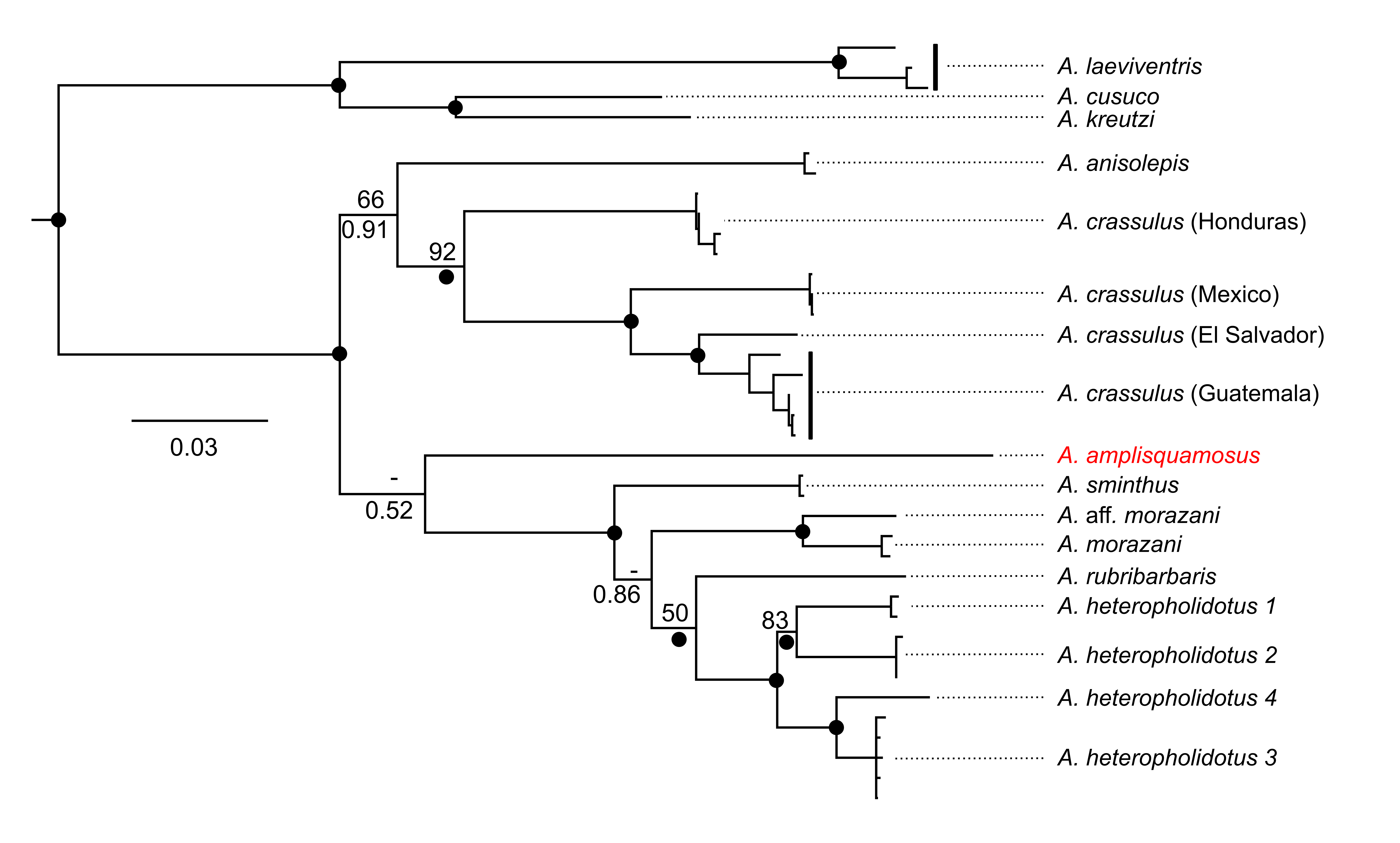

Supplement: Supplementary file 2 — Phylogenetic hypothesis of the Anolis crassulus species subgroup based on a concatenated datset of six loci analysed in RAxML and MrBayes. Nodes are labelled with maximum likelihood bootstrap support (BS; above), and Bayesian inference posterior probabilities (PP; below); Black circles indicate BS ≥ 95 or PP ≥ 0.95, and a single black circle on the node indicates both BS & PP ≥ 95 & 0.95, respectively. BS < 50 and PP < 0.50 not shown. Population names in red are those not recovered in an identical position to the StarBEAST2 species tree (Fig. 4). (TIFF 4374 kb) [file 12862_2017_1115_MOESM2_ESM.tif]
